# Sepsis 2019: Rio de Janerio, Brazil. 09 - 10 May 2019

**DOI:** 10.1186/s40635-019-0254-1

**Published:** 2019-06-27

## Sepsis 2019 Abstracts

### P1

**Withdrawn**

### P2 Epigenetic changes are reported in animal model of sepsis

#### Monique Michels^1^, Mariane Rocha Abatti^1^, Andriele da Silva Vieira^1^, Heloisa Borges^1^, Amanda Indalécio Goulart^1^, Roger Varella^2^, Samira Valvassori^2^, Felipe Dal-Pizzol^1^

##### ^1^Laboratory of Experimental Pathophysiology, Extreme University South of Santa Catarina, Criciúma, Brazil; ^2^Laboratório de Neurociências, Programa de Pós-Graduação em Ciências da Saúde (PPGCS), Universidade do Extremo Sul Catarinense (UNESC), Criciúma, SC, Brazil

###### **Correspondence:** Monique Michels (moniquemichels@hotmail.com)

Background

The presence of oxidative stress and inflammatory mediators in sepsis may lead to epigenetic changes [1,2]. Epigenetic alterations of histones, such as methylation, acetylation and phosphorylation may direct the folding or unfolding of DNA through mechanisms still unknown, thus altering gene transcription [3]. Once gene transcription in sepsis is altered the response may be further exacerbated. Our objective was to report epigenetic changes in brain structures in animal model of sepsis.

Materials and Methods

Male Wistar rats were subjected to sham or CLP and cerebral structures were removed in 24h, 72h, 10, 30 and 60 days after sepsis. HAT, HDAC and DNMT enzymes activities were measured in frontal cortex and hippocampus in different times.

Results

No changes found in HAT activity (Fig. 1). Increased HDAC (Fig. 2) and DNMT activity (Fig. 3) was observed 72h, 10 and 30 days after sepsis and a significant reduction 60 day after.

Conclusions

It’s possible observe epigenetic alterations and deregulation in gene transcription in animal model of sepsis. Since in sepsis the presence of oxidative stress and the release of inflammatory mediators are well reported, these insults can lead to epigenetic changes, such as gene transcription, which may be related to exacerbation of the inflammatory response. Environmental influences can modulate the epigenetic response and therefore, be a therapeutic strategy for the treatment of sepsis.

References

1. Margueron R, Trojer P, Reinberg D: The key to development: interpreting the histone code? Curr Opin Genet Dev. 2005, 15:163–176.

2. Reik W: Stability and flexibility of epigenetic gene regulation in mammalian development. Nature. 2007, 447:425–432.

3. Chen Y, Hong T, Wang S, Mo J, Tian T, Zhou X: Epigenetic modification of nucleic acids: from basic studies to medical applications. Chem Soc Rev. 2017, 46(10):2844-2872.

**Fig. 1 (abstract P2).**
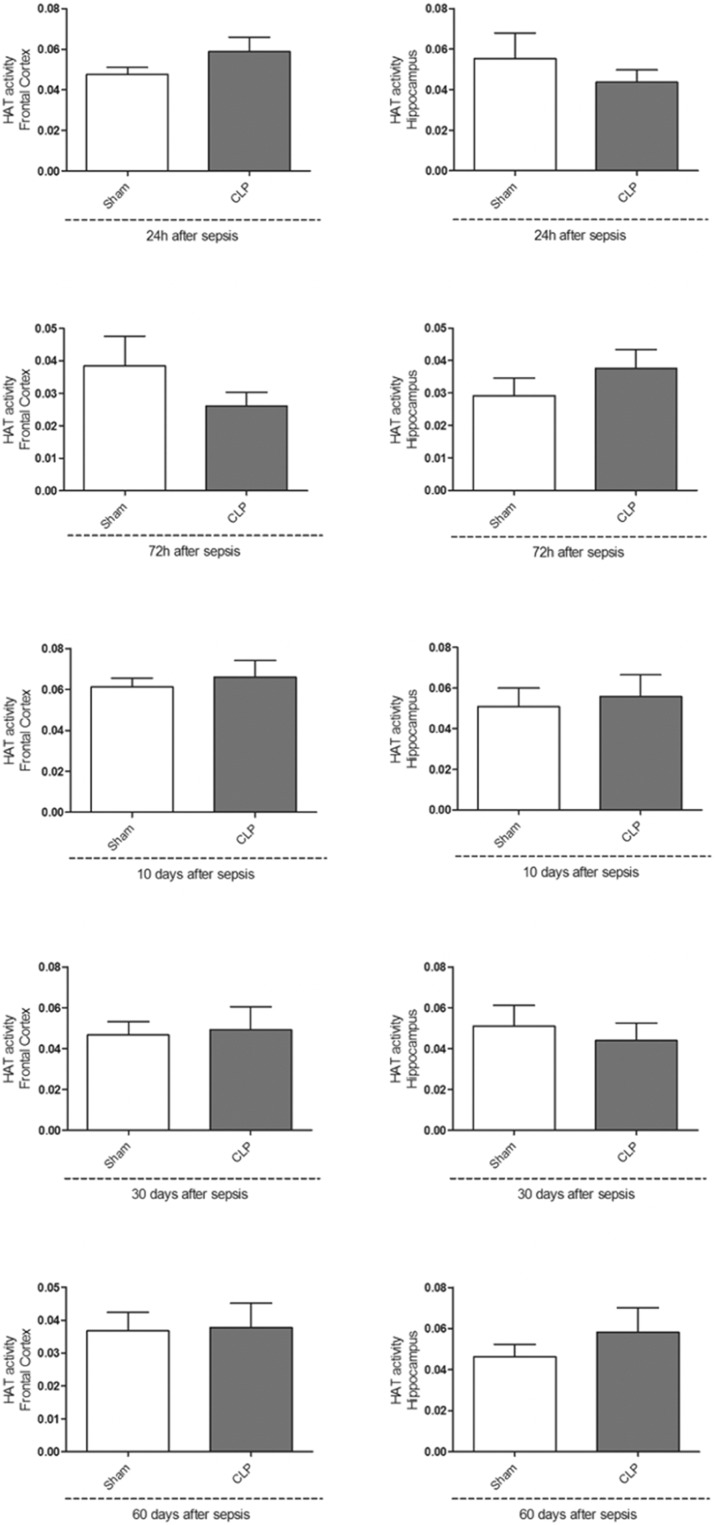
See text for description

**Fig. 2 (abstract P2).**
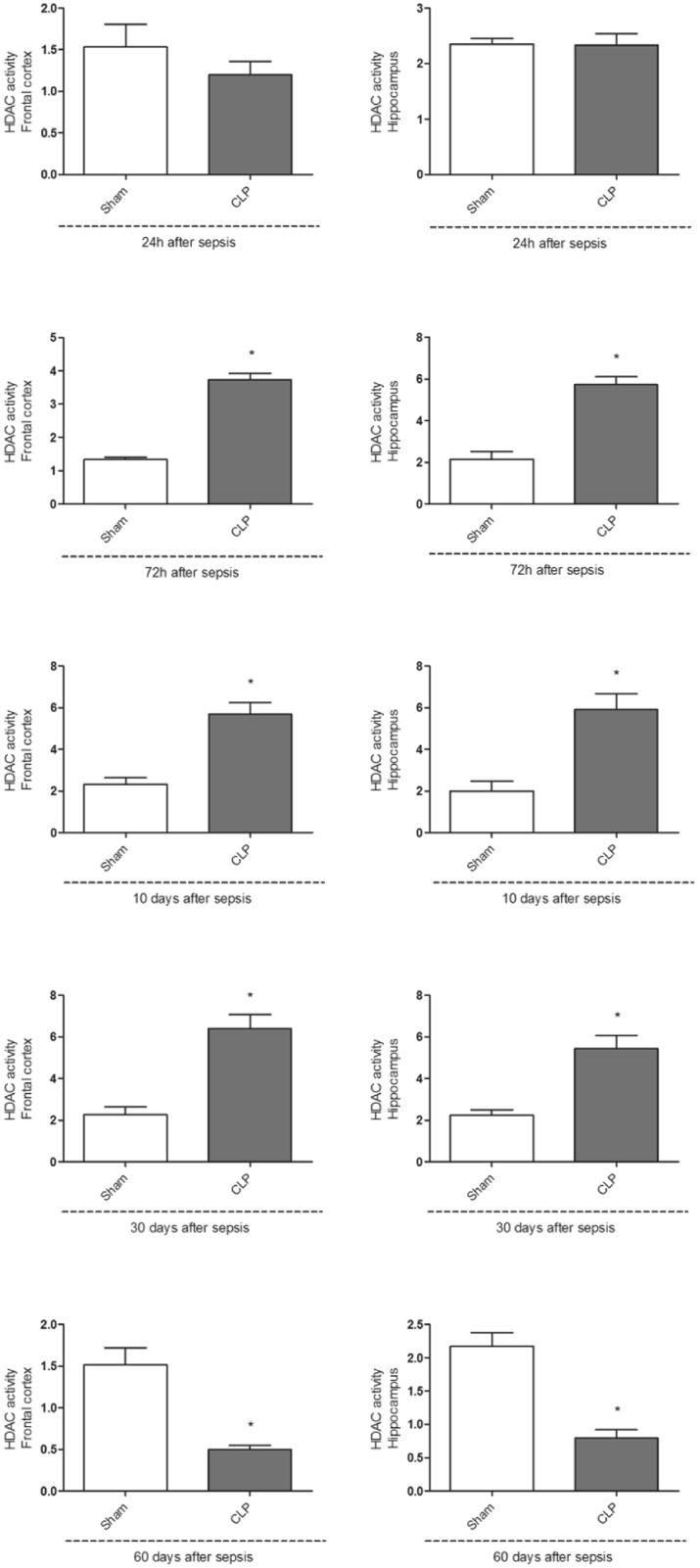
See text for description

**Fig. 3 (abstract P2).**
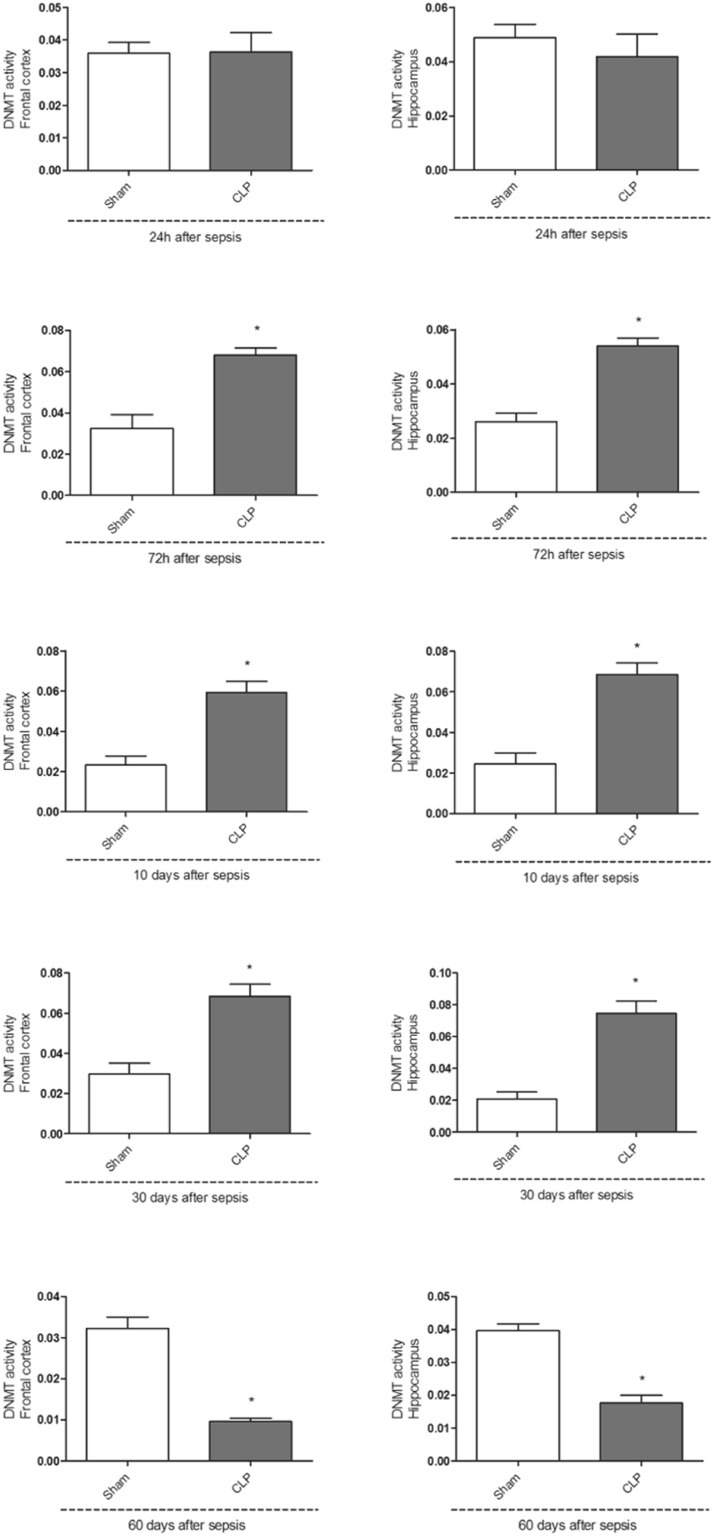
See text for description

### P3 Is antibiotic treatment always necessary for chronic critical ill patients?

#### Natalia Beloborodova, Irina Buyakova, Ekaterina Chernevskaya

##### Federal Research and Clinical Center of Intensive Care Medicine and Rehabilitology, Laboratory of metabolism in critical state, Moscow, Russia

###### **Correspondence:** Ekaterina Chernevskaya (chea05@inbox.ru)

Background

Antibiotics are prescribed to almost all of chronic critical ill (CCI) patients. This often leads to colonization of multi-resistant strains of microorganisms and dramatic disturbances of gut microbiota. Metabolic activity of microbes can be assessed by the measurement of the levels of aromatic microbial metabolite (AMM) in serum, which are associated with the severity and mortality of ICU patients [1]. The modern trend is to reduce the antibacterial pressure. The aim of our study is to estimate the frequency of antibiotic use in CCI patients, to discuss the relationship between change in the neurological status and metabolic profile of AMM.

Materials and Methods

The study included 40 CCI patients with neurological disorders (stroke, traumatic brain injury, neurosurgical intervention for brain tumors). The level of AMM was measured in blood serum using GC-MS (Thermo Scientific). Biomarkers (PCT, S100) were measured using Elecsys immunoassay.

Results

Antibiotics (cephalosporins, aminoglycosides, fluoroquinolones, etc.) were prescribed on 74% of CCI patients in different cases (bacteriuria, leukocyturia, fever, etc.), but the retrospective analysis showed that levels of PCT were low (0.02 to 0.236 ng/ml). A significant increase the level of AMM, mainly due to para-hydrophenylacetic acid (p-HPhAA) was accompanied by the negative dynamics of the somatic and neurological status. Low serum levels of AMM correlated with positive clinical dynamics, a decrease of p-HPhAA level and an increase in para-hydroxybenzoic acid correlated with an improvement in neurological status. AMM`s data are compared with analysis of the gut microbiota using 16S rRNA sequencing in different groups of patients.

Conclusions

In chronic critical ill patients with neurological disorders, the presence of indirect signs of infection is not an indication for antimicrobial therapy, if biomarkers and AMM remain within the reference values, since antibiotics do not contribute to the improvement of the clinical and neurological condition. For this group of patients’, it is necessary to develop new treatment strategies based on the correction of microbiota metabolism.

References

1. Beloborodova NV, Sarshor YN et al.: Involvement of Aromatic Metabolites in the Pathogenesis of Septic Shock. Shock. 2018, 50(3):273-279.

### P4 Connection between biomarkers and aromatic metabolites in сerebrospinal fluid in critically ill patients

#### Ekaterina Chernevskaya^1^, Natalia Beloborodova^1^, Tatyana Litvinova^1^, Irina Alexandrova^2^, Maria Getsina^1^, Alisa Pautova^1^

##### ^1^Federal Research and Clinical Center of Intensive Care Medicine and Rehabilitology, Laboratory of metabolism in critical state, Moscow, Russia; ^2^Federal State Autonomous Institution “N .N. Burdenko National Scientific and Practical Center for Neurosurgery”, Moscow, Russia

###### **Correspondence:** Ekaterina Chernevskaya (chea05@inbox.ru)

Background

Some aromatic derivatives of tryptophane - 5-hydroxyindoleacetic acid (5-HIAA) and 3-indoleacetic acid (3-IAA) are under direct or undirect control of the gut microbiota and may play an important role in gut–brain axis. It was found that in serum high levels of some aromatic microbial metabolites (AMM) of phenolic structure are associated with severity of infection in critically ill patients with sepsis. Neuron specific enolase and S100 protein are biomarkers that reflect the neurotrophic and neurotoxic effects of neuron and glial cells. Procalcitonin (PCT) is a biomarker which is elevated in serum in the case of bacterial infection, however, the usefulness of PCT measurement in CSF has shown conflicting results. Cut-off value of PCT in CSF for bacterial meningitis was lower than serum level of PCT [1]. High level of PCT in CSF may indicate the loss of integrity of the blood-brain barrier, but some microbial metabolites may also penetrate the barrier in critical conditions. The aim of our study was to identify and qualify indolic and phenolic metabolites and evaluate correlation of certain biomarkers in the CSF in critically ill patients.

Materials and Methods

The study included 37 CSF samples taken from neurosurgical critically ill patients with CNS infection, traumatic brain injury. The levels of AMM and 3-IAA were measured in the CSF using GC-MS (Thermo Scientific), biomarkers (PCT, S100, NSE, IL6) were measured by Elecsys immunoassay, 5-HIAA was measured by ELISA (Cloud-Clone Corp).

Results

The median level of the sum of 6 aromatic metabolites (benzoic, phenyllactic (PhLA), p-hydroxybenzoic (p-HBA), p-hydroxyphenilacetic, homovanilic (HVA) and p-hydroxyphenillactic (p-HPhLA) acid) in the CSF was 4.4 μM. The direct Spearman`s correlation between the level of PCT and sum of AMM (0.35, p<0.05) was revealed. The correlations between neurological biomarkers and some phenolic and indolic metabolites were also revealed (table 1).

Conclusions

Connection between aromatic metabolites and neurological biomarkers (S100, NSE) indicates the potential involvement of phenolic and indolic metabolites in the pathogenesis of brain dysfunction.

Acknowledgements

Supported by the Russian Science Foundation Grant 15-15-00110

Reference

1. Zhang L, Ma L et al. Diagnostic Value of Procalcitonin for Bacterial Meningitis in Children: A Comparison Analysis Between Serum and Cerebrospinal Fluid Procalcitonin Levels. Clin Pediatr (Phila). 2018 Oct 29:9922818809477

**Table 1 (abstract P4).** Correlations between biomarkers and aromatic metabolites (p<0.05)

Parameter	РСТ, ng/ml	S100, mkg/l	NSE, ng/ml	5-HIAA, ng/ml	3-IAA	Phenolic metabolites, μM				
						PhLA	p-HBA	HVA	p-HPhLA	Sum
IL6, pg/ml	ns	ns	ns	**0.39**	ns	**0.37**	ns	ns	ns	ns
РСТ, ng/ml	ns	**0.33**	**0.37**	ns	ns	ns	ns	ns	ns	**0.35**
S100, mkg/l	**0.33**	ns	ns	ns	ns	ns	**0.39**	**0.42**	ns	ns
NSE, ng/ml	**0.37**	ns	ns	**0.43**	**0.36**	ns	**0.50**	ns	**0.36**	ns

### P5 Association between site of infection and in-hospital mortality among patients with sepsis in the emergency departments of third-level hospitals in Medellin, Colombia

#### César Caraballo^1,4^, Johana Ascuntar^1^, Carolina Hincapié^1^, Camilo Restrepo^1^, Elisa Bernal^2^, Fabián Jaimes^1,3^

##### ^1^Grupo Académico de Epidemiologíıa Clínica (GRAEPIC), Universidad de Antioquia, Medellín, Colombia; ^2^Servicio de Medicina Interna, Hospital Pablo Tobón Uribe, Medellín, Colombia; ^3^Dirección de Investigaciones, Hospital San Vicente Fundación, Medellín, Colombia; ^4^Center for Outcomes Research and Evaluation (CORE), Yale University School of Medicine, New Haven, CT, USA

###### **Correspondence:** Fabián Jaimes (fabian.jaimes@udea.edu.co)

Background

The impact of infection site in patients with sepsis on hospital mortality have not been reliably estimated1. We aimed to determine, in patients presenting to the emergency department with sepsis or septic shock, the association between the infection site and in-hospital mortality.

Materials and Methods

Multicenter prospective cohort in three emergency departments and critical care units of high complexity hospitals in Medellín (Colombia). We recruited patients older than 18 years admitted with sepsis or septic shock as the main diagnosis. The exposure variable was site of infection according to standardized CDC definitions and the primary outcome variable was in-hospital mortality. A hierarchical logistic regression model was fitted for adjusting for acknowledged prognostic factors as comorbidities, organ dysfunction and emergency treatments.

Results

From 5022 eligible patients, 2510 were included in the study. The most frequent site of infection was the urinary tract with 27.8% of the cases, followed by pneumonia with 27.5% and intra-abdominal focus with 10.8% of the patients. In the 5.4% of the cases there was not a clear site of infection at admission. Using hierarchical logistic regression models with urinary tract as the reference, there were significant differences in mortality (Table 1).

Conclusions

There is an association between the different sites of infection and in-hospital mortality in patients with sepsis and septic shock and this should be considered in the prognostic models for these conditions.

Reference

1. Motzkus CA, Luckmann R. Does infection site matter? A systematic review of infection site mortality in sepsis. J Intensive Care Med. 2017;32:473-9.

**Table 1 (abstract P5).** Hierarchical Logistic regression and mortality

Site of infection	Univariate	Multivariable^1^	Multivariable^2^	Multivariable^3^
	OR (95%CI)	OR (95%CI)	OR (95%CI)	OR (95%CI)
Urinary tract	1.0	1.0	1.0	1.0
Lower airway	4 (2.7 - 6)	3.8 (2.6 - 5.8)	3.9 (2.6 - 5.8)	3.4 (2.2 - 5.2)
Intra-abdominal	3.1 (1.9 - 5)	3.2 (1.9 - 5.1)	3.1 (1.9 - 5.0)	1,9 (1.1 - 3.3)
Skin and soft tissues	1.6 (0.9 -2.9)	1.8 (0.9 -3.2)	1.8 (1.0 - 3.2)	2.6 (1.4 - 5.0)
Bloodstream	3.3 (1.9 - 5.5)	3.4 (2 - 5.7)	3.2 (1.9 - 5.3)	2.0 (1.1 - 3.6)
Unknown	3.5 (2.0 -6.2)	3.4 (1.9 -6.1)	3,3 (1,9 - 5,9)	2,0 (1,1 - 3,8)
Other	1.9 (1.1 – 3.2)	2 (1.2 - 3.5)	2 (1.1 - 3.4)	1.5 (0.8 - 2.8)

^1^Adjusted for age, sex, Charlson

^**2**^Adjusted for model 1 plus IVF > 1500 first hour, antibiotics and blood cultures first three hours

^**3**^Adjusted for model 2 plus lactate, SOFA and APACHE II Scores

### P6 Risk factors for development of sepsis in a pediatric ICU

#### Gabriel do Amaral Cavalcante¹, Andrea Ramires Kairala², Rafael Augusto Faust Machado³, Bruno Mamede Lins Brasiliense³, Gabriela Jordão Vieira Gomes^4^

##### ¹Medicine department of UniCEUB, Brasília, Brasil; ²Instituto Hospital de Base, Brasília, Brasil; ³Brasil medicine department of UNICEPLAC, Brasília, Brasil; ^4^Secretaria de Estado de Saúde do Distrito-Federal, Brasília, Brasil

###### **Correspondence:** Gabriel do Amaral Cavalcante (cavalcante.g@gmail.com)

Objective

To determine and evaluate clinical aspects and the main risk factors for development of sepsis in patients admitted in a pediatric Intensive Care Unit (ICU).

Materials and methods

We performed a retrospective and observational study by evaluating electronic medical records of patients, aged between 0 and 12 years old, admitted among January and December of 2017 in a pediatric ICU at a tertiary care hospital in Distrito-Federal. Patients that died in 48 hours of the admission, immunocompromised or admitted in the unit already with sepsis were excluded.

Results

Three hundred and thirty-four children were admitted in 2017, 171 of those were included in the study. The majority were male (N: 102; 59.6%) and under 2 years old (N:107;62.6%). 16 died (9.4%). Most of them hospitalized for 6 to 12 days (N:67; 39.2%). The reason of the hospitalization were clinical (N:92; 53.8%), surgical (N:72; 42.1%) and trauma (N:7; 4.1%). Central venous access was made in 136 (78.9%) patients, followed by tracheal intubation (N:109; 63.7%), thorax drainage (N:20; 11.7%) and 50% had long-standing bladder catheter. In our sample, 52 (30.4%) were diagnosed with sepsis and, among those, 8 evolved to septic shock. 24 (46.2%) of whom with sepsis had age between 28 days and 2 years. Also among those with sepsis, 75% had tracheal intubation, there being statistical correlation between the procedure and sepsis (p-value: 0.043). The other procedures didn’t show correlation with the development of sepsis. The sites of the infection among those with sepsis were mainly pulmonary (67%), central nervous system (13%), urinary tract (3%), abdominal (8%), cardiovascular (3%) and others (6%). 5 patients with diagnosis of sepsis died (9.6%). There was found no relation between the diagnosis of sepsis and age, sex and death, (p-value = 0.758) (p-value = 0.995) and (p-value = 0.939) respectively.

Conclusions

In a pediatric ICU, besides the medical reason of the hospitalization, there are necessary invasive procedures that contribute to the development of infections of many levels; there are mechanisms of protection and investigation that can lower sepsis incidence, also leading to an inferior death risk and incidence of complications during the staying in hospital.

### P7 Early sepsis diagnosis: lesser internship time and death in a pediatric ICU

#### Gabriel do Amaral Cavalcante¹, Andrea Ramires Kairala², Rafael Augusto Faust Machado³, Bruno Mamede Lins Brasiliense³, Gabriela Jordão Vieira Gomes^4^

##### ¹Medicine department of UniCEUB, Brasília, Brasil; ²Instituto Hospital de Base, Brasília, Brasil; ³Medicine department of UNICEPLAC, Brasília, Brasil; ^4^Secretaria de Estado de Saúde do Distrito-Federal, Brasília, Brasil

###### **Correspondence:** Gabriel do Amaral Cavalcante (cavalcante.g@gmail.com)

Objective

To demonstrate the importance of early diagnosis and treatment in patients with sepsis in a pediatric intensive care unit (ICU).

Materials and methods

A retrospective observational study by evaluating electronic medical records of patients, aged between 0 and 12 years old, admitted in 2017 in a pediatric ICU at a tertiary care hospital. Patients that died in 48 hours since admission, immunocompromised or already admitted with the diagnosis of sepsis were excluded.

Results

Three hundred and thirty-four children were admitted in 2017, 171 were included in the study. The majority were male (N: 102; 59.6%) and under 2 years old (N:107;62.6%). 16 died (9.4%). Most were hospitalized for 6 to 12 days (N:67; 39.2%). The reason of the hospitalization was clinical (N:92; 53.8%), surgical (N:72; 42.1%) and trauma (N:7; 4.1%). 52 (30.4%) patients were diagnosed with sepsis. 24 (46.2%) of whom with sepsis were aged between 28 days and 2 years old. 5 (9.6%) of them died, which means 2.9% of total patients admitted in the ICU and 31% of total death registered in the unit in 2017 (p-value = 0.033), therefore showing correlation between death and sepsis. The average time from admission and diagnosis was 3.6 days; done in < 5 days in 43 patients. Septic shock was identified in 8 patients, the diagnosis time was < 5 days in all of them. Those with sepsis (N:25; 48.1%) remained > 13 days in the unit; those without sepsis (N:86; 72.2%) remained <13 days in the unit (p-value = 0.025). With regard to the PIM score, patients with sepsis (19.4%) had it between 0 to 1,1; and among those without sepsis (34.5%) were at the same score range (p-value = 0.025). Therefore showing correlation of higher scores and the possibility of the sepsis diagnosis.

Conclusions

Sepsis is a serious public health issue; the diagnosis is clinical and the faster and aggressive the treatment begins, it alters drastically the prognosis and development of the disease. The primary objective of the first hours of diagnosis is lower the death risk and restore signs of hypoperfusion, lowering the death risk and time of internship.

### P8 Evaluation of pathogenic agents and antimicrobial susceptibility of chronic suppurative otitis media at Kigali Universality teaching hospital

#### Marie F Kayitesi^1^, Claude M Muvunyi^2^, Evariste Mushuru^3^, Rajab Mugabo^4^

##### ^1^ENT department, Butare University teaching hospital, Butare, Rwanda; ^2^Microbiology Department, National referral laboratory, Kigali, Rwanda; ^3^Internal Medicine department, Butare University teaching hospital, University of Rwanda, Butare, Rwanda; ^4^ENT Department King Faisal hospital, Kighali, Rwanda

###### **Correspondence:** Marie F Kayitesi (mfkayitesi@gmail.com)

Background

Chronic suppurative otitis media is a chronic inflammation of the middle ear and mastoid cavity, with more than 2 weeks of otorrhea. Various studies have shown that both gram-positive and gram-negative bacteria, which differ according to the sites, are responsible for infection of middle ear. The knowledge of the prevailing flora and their susceptibility to antibiotics is an important step for an appropriate treatment

Materials and Methods

The current study was cross sectional survey involving enrolled 110 patients who consulted ENT Department at KUTH with active chronic suppurative otitis media or its complication, from November 2014 up to January 2015. The patient demographics, clinical presentation, microbiology and antibiotic sensitivity were collected using data collection sheet.

Results

The age of our population ranged between 2 and 89 years, the maximum was in the age range of 16- 30 years (55.5%). The proportion of male to female was almost similar, male constituted 50. 9% while females were 49.1%. The majority had discharge for more than 5 years. For the results of culture and sensitivity, 65.5% showed significant microbial growth of single organism, with majority being *Staphylococcus aureus* 35%, followed by *Klepsiella spp* 15%, and Pseudomonas aeruginosa together with *Enterobacter spp* accounting for 10 % for each. *S.aureus* showed high sensitivity to ciprofloxacin and clindamycin, but it were resistant to penicillin. For overall of antimicrobial used, ciprofloxacin was revealed to be most effective antimicrobial drug against many organisms at 51.8%. Chloremphenicol was effective at 14.5% while cefotaxim and augmentin showed to be effective at 10% and 8.2% respectively.

Conclusions

There is variation in isolated organisms as well as antimicrobial drugs. For this reason, to know the exact sensitive antibiotic to a certain ear infection treated without success, it is advisable to do culture of discharge and sensitivity.

### P9

**Withdrawn**

### P10 Etiology and antimicrobial resistance patterns of neonatal sepsis at Mulago National Referral Hospital, Uganda

#### Josephine Tumuhamye^1,4^, Halvor Sommerfelt^1^, Freddie Bwanga^2^, James K Tumwine^3^, David Mukunya^1,2^, Victoria Nankabirwa^1,4^

##### ^1^Centre for Intervention Science in Maternal and Child health (CISMAC) and Centre for International Health, University of Bergen, Norway; ^2^Department of Medical Microbiology, Makerere University Kampala, Uganda; ^3^Department of Pediatrics and Child Health, Makerere University Kampala, Uganda, ^4^Department of Epidemiology and Biostatistics, Makerere University Kampala, Uganda

###### **Correspondence:** Josephine Tumuhamye (tphynne@gmail.com)

Background

Globally, approximately 2.5 million babies die in the first month of life[1]. Nearly all (99%) of these neonatal deaths occur in low income countries. The aim of this study was to describe the bacterial etiology and the antimicrobial resistance patterns of the isolated bacteria among newborns clinically suspected of having sepsis

Materials and Methods

A cross-sectional study was conducted at the Mulago national referral hospital in Kampala, Uganda. Venous blood for culture was collected from 305 newborns with clinical signs of sepsis. Validated questionnaires on mobile devices were used to obtain sociodemographic characteristics. An automated blood culture system was used (BD BactecTM) plus other conventional culture methods. Kirby Bauer disk diffusion method was used for antimicrobial susceptibility testing according to clinical laboratory standard institute. mecA PCR was conducted for confirmation of methicillin resistant *Staphylococcus aureus* (MRSA)

Results

The mean birth weight of the neonates was 3.1 kg (SD 0.6), 32% of them were ≤7 days old and 55% were males. The proportion of patients with a bacterial pathogen known to cause sepsis was 14% (95% CI; 10%-19%). This included 27 *Staphylococcus aureus* isolates, *Escherichia coli* (6), *Klebsiella pneumoniae* (5), *Streptococcus pneumoniae* (1), *Neisseria spp* (1), *Enterobacter spp* (1) and *Citrobacter freundii* (1). All the 5 *K.pneumoniae* isolates, 5/6 *E.coli* isolates and 26/27 *S.aureus* isolates were resistant to ampicillin. Resistance to the most commonly used aminoglycoside varied between species in that 6 (22%) of the *S. aureus*, one of the *E. coli* and two of the K. pneumoniae isolates were resistant to gentamicin. Among the twenty seven *S.aureus* isolated, 20(74%) were MRSA, 19 (70%) were resistant to erythromycin, 10 (37%) were resistant to ciprofloxacin, 7 (26%) were resistant to trimethroprim-sulphamethoxazole and 8(30%) displayed erythromycin inducible clindamycin resistance (D-test positive). However all *S.aureus* isolates were sensitive to vancomycin. Three Gram-negative enteric bacterial isolates were extended broad spectrum beta lactamase producers; 1 *E. coli* and 2 *K. pneumoniae* but were sensitive to imipenem.

Conclusions

*S. aureus* was the most common bacterial isolate among newborns with clinical signs of sepsis at the national referral hospital. The high frequency of MRSA among these isolates is worrisome and questions the empirical management of neonatal sepsis. Erythromycin inducible clindamycin resistance further limits treatment options for MRSA infections

Reference

1. Liu L, Oza S, Hogan D, Perin J, Rudan I, Lawn JE, et al. Global, regional, and national causes of child mortality in 2000-13, with projections to inform post-2015 priorities: an updated systematic analysis. Lancet (London, England). 2015;385(9966):430-40.

### P11 Elevated levels of Nt-proBNP, proinflamatory cytokines, procalcitonin and lactate are associated with increased risk of mortality in Sepsis and Acute Renal Injury patients

#### Luis Huespe, Silvio Lazzeri, Carlos Mizdraji, Liu Ting, Santiago Ballejos, Lara Costa, Fabian Plano, Juan Melana, Tania Stoyanof, Victoria Aguirre, Monica Auchter, Juan Pablo Rodríguez

##### Intensive Care Unit, San Martín University Hospital and Biomolecular Research Laboratory, Faculty of Medicine. UNNE Rivadavia 1250 Corrientes (3400)-Argentina

###### **Correspondence:** Luis Huespe (dythe_hescuela@hotmail.com)

Background

Sepsis is a potentially fatal organ dysfunction caused by a dysregulated host response to infection. Acute kidney Injury is the most frequent complication in patients with septic shock and is an independent risk factor for death. Patients diagnosed with Sepsis-3 were included in a prospective observational protocol with the following objectives: 1) Mortality at 28 and 90 days, 2) Acute Renal Injury and causes of non-recovery at 7 days and 3) Type-5 Cardiorenal Syndrome.

Materials and Methods

All patients with Sepsis-3 were were included in the study (December 2017-December 2018.) Epidemiological data, SOFA, Nt-proBNP, proinflamatory cytokines, procalcitonin, lactate, primary site of infection, microbiological culture, days of ventilación and standard care were determined. To identify the subgroup of patients with ARF, we used sepsis as an initial insult and the KDIGO criteria to determine creatinine increase ≥ 0.3 mg / dl or 50% of the previous lower value within 48 hours of admission to the protocol, or urine volume <0.5 ml/kg/H in the same period. Patients with CKD or hemodialysis before admission were excluded.

Septic shock was established in the initial protocol and NA drugs (5 μg/minute) were administrated. Aditionally, Nt-proBNP and echocardiographic were determined. Blood samples were collected and mRNA of proinflammatory cytokines were measured by RT-qPCR.

Results

From all patients (n=385) admitted to ICU, 54 of them were diagnosed with Sepsis-3. Average data showed: Age 44.2 years (20-81y), SOFA 7.3 (2-14), Nt-proBNP 5610 pg/dl (112-21890), Procalcitonin 17,68 ng/dl (0.7-100), Lactate 2.45 mmol (1.69-10.7) Ventilation 8,9 days. Patients with KDIGO criteria 22 of which 5 patients (18%) required hemodialysis, 18 patients (33.3%) had an Nt-proBNP> 1000 and mortality was 40%. If we compare it to this subgroup over the totality of the annual patients, mortality was 3.63%, renal replacement therapy 1.29%, septic shock 14.02% and sepsis-3 22.07%. The use of vasoactive drugs 10.03% and Cardiorenal Syndrome type-5 was 8.05%. All patients have elevated levels of interleukins 6,7,10 and 12

Conclusions

Acute Kidney Injury and non-recovery at seven days after the initial insult in patients with sepsis and septic shock increases the mortality at 90 days. The identification of a subgroup of patients is useful for directing therapeutics and biomarker determinations are necessary. The association of renal involvement and transient cardiac failure can make us suspect the presence of type 5 cardiorenal syndrome.

### P12 Serum-induced cytotoxicity of patients with sepsis in cell culture HEK- Preliminary results for the development of a rapid AKI diagnostic test

#### Luis Huespe, Silvio Lazzeri, Carlos Mizdraji, Diego Farizano, Rodrigo Sanabria, Juan Melana, Tamara Barnes, Victoria Aguirre, Juan Todaro,Roberto Jabornisky, Monica Auchter, Juan Pablo Rodriguez

##### Intensive Care Unit, San Martín University Hospital and Biomolecular Research Laboratory, Faculty of Medicine. UNNE Rivadavia 1250 Corrientes (3400), Argentina

###### **Correspondence:** Luis Huespe (dythe_hescuela@hotmail.com)

Background

Sepsis is a potentially fatal organ dysfunction caused by a dysregulated host response to infection. Acute kidney Injury is the most frequent complication in patients with septic shock and we hypothesize that the damage is due to toxins related to infection, which is why we use in vitro cultures with HEK -293 cell.

Materials and Methods

All patients with Sepsis-3 were included in the study (December 2017-December 2018.)

Patients with ARF, we used sepsis as an initial insult and the KDIGO criteria to determine AKI. Blood samples from patients Sepsis.3 (n 15) were obtained with the prior informed consent and the bioethical standards of the Hospital Committee. The biochemical tests were analyzed in quadruplicate and *in vitro* test using HEK-293 cell line, in a humid atmosphere with 5% C02 and 37°C. Cell monolayer was grown up to 60% confluence using RPMI with 5% fetal bovine serum. Once the monolayers were obtained, culture medium was removed and washed 3 times with 1X PBS. Inverted optical microscopy was used at different magnifications 40-400X

Results

From all patients (n=385) admitted to ICU, 54 p with Sepsis-3. Average data showed: Age 44.2 years (20-81y), SOFA 7.3 (2-14), p with KDIGO criteria 22 of which 5 patients (18%) required hemodialysis, 18 patients (33.3%) had an Nt-proBNP> 1000 and mortality was 40%. 15 p of them were analyzed in vitro test using HEK-293 cell line and subsequently, cells were treated as the following: A HEK-293 culture with incomplete RPMI medium (control without fetal bovine serum), B HEK-293 cell treated with non-septic serum, C HEK-293 cell treated with septic serum in concentrations (0.5%, 2.5%, 5%, 10% and 15%). Cell cultures incubated for 24 hs, and morphological analysis for monolayers to evaluate changes compatible with cellular death. Control cell grown in incomplete RPMI medium cells treated with non-septic serum did not show significant differences between them and no cytoplasmatic or nuclear damage was observed. However, treated cells showed cell damage in direct relation to the amount of septic serum added to the culture.

Conclusions

Acute Kidney Injury and non-recovery at seven days after the initial insult in patients with sepsis and septic shock increases the mortality. This group demonstrate *in vitro* damage caused by cytoxin from the infectious focus. This result lead us to think that the development of a rapid technique to diagnose AKI with a cell culture laboratory.

### P13 The effect of Intelligent Sepsis Management System on survival outcome in patients with sepsis and septic shock in accordance with Sepsis-3 definitions

#### Juhyun Song^1^, Daewon Park^2^, Sungwoo Moon^1^, Hyeri Seok^2^, Sejoong Ahn^1^

##### ^1^Emergency Department, Korea University Ansan Hospital, Republic of Korea; ^2^Infectious Diseases, Korea University Ansan Hospital, Republic of Korea

###### **Correspondence:** Juhyun Song (songcap97@hotmail.com)

Background

Sepsis is a global public health problem representing a leading cause of morbidity and mortality and increased costs in many countries [1]. According to a previous study, implementation of a national sepsis program resulted in improved adherence to sepsis bundles in severe sepsis and septic shock patients and was associated with reduced adjusted in-hospital mortality [2]. Another recent study reported that implementation of multidisciplinary emergency department (ED) sepsis bundle was associated with improved time to achieve key therapeutic interventions and a reduction in 30-day mortality [3]. In other study, the use of a sepsis triage screening tool significantly decreased the time to antibiotics in patients presenting to the ED [4]. Surviving Sepsis Campaign (SSC) 2016 recommends that hospitals have a performance improvement program for sepsis [5]. We have newly developed Intelligent Sepsis Management System (i-SMS) which help clinicians to screen, diagnose, and manage septic patients. The purpose of the present study was to assess the effect of i-SMS on compliance with the SSC bundles and survival outcome in patients with sepsis and septic shock diagnosed in ED. In addition, we tried to determine risk factors for 28-day mortality.

Materials and methods

We performed a pre-post study in patients with sepsis and septic shock. During the pre-period (January 1, 2016-Setember 25, 2017), patients were managed with routine customary process. During the post period (September 26, 2017-July 10, 2018), patients were managed with assistance from i-SMS upon arrival to ED.

Results

A total of 548 patients were included; 317 in preperiod and 231 in postperiod (Table 1 and Figure 1). After implementation of i-SMS, overall compliance with SSC recommendations improved from 26.8% to 52.8% (P <0.001) (Figure 2). There was no significant difference in 28-day mortality between preperiod (37.2%) and postperiod (32.0%) (P = 0.08) (Table 1). In Kaplan-Meier survival analysis and Log-rank test, there was no significant difference of survival curves between preperiod and postperiod (P = 0.666) (Figure 3). SOFA score and lactate levels were independent risk factors for 28-day mortality in all enrolled patients (Table 2).

Conclusions

Implementation of i-SMS significantly improved compliance with SSC recommendations among patients with sepsis and septic shock in accordance with Sepsis-3 definitions, but did not improve short-term survival outcome.

Acknowledgements

The authors thank research nurse Hye-Yoon Jung and researcher Min-Sook Jung for their contributions to the project. We also thank Jae-Hyung Cha, PhD for lending statistical

support.

References

1. Angus DC and van der Poll T. Severe sepsis and septic shock. N Engl J Med. 2013;369(9):840-51.

2. Van Zanten AR, Brinkman S, Arbous MS, Abu-Hanna A, Levy MM, de Keizer NF. Guideline bundles adherence and mortality in severe sepsis and septic shock. Crit Care Med. 2014;Aug;42(8):1890-1898

3. McColl T, Gatien M, Calder L, Yadav K, Tam R, Ong M, Taljaard M, Stiell I. Implementation of an Emergency Department Sepsis Bundle and System Redesign: A Process Improvement Initiative. CJEM. 2017 Mar;19(2):112-121

4. Patocka C, Turner J, Xue X, and Segal E. Evaluation of an emergency department triage screening tool for suspected severe sepsis and septic shock. J Health Qual. 2014 Jan-Feb;36(1):52-61

5. Rhodes A, Evans LE, Alhazzani W, Levy MM, Antonelli M, Ferrer R, Kumar A, Sevransky JE, Sprung CL, Nunnally ME, et al. Surviving Sepsis Campaign: International Guidelines for Management of Sepsis and Septic Shock:2016. Crit Care Med. 2017; 45(3):486-552.

**Table 1 (abstract P13).** Baseline characteristics of enrolled patients and comparison between preperiod and postperiod

	Pre (N = 317)	Post (N = 231)	P value
Age (years), median (IQR)	71 (60-79)	73 (62-82)	0.148
Male, no. (%)	158 (49.8)	115 (49.8)	0.996
Initial q-SOFA ≥2, no. (%)	317 (100)	231 (100)	
Site of infection, no (%)			
Respiratory	198 (62.5)	135 (58.4)	0.315
Genitourinary	83 (26.2)	61 (26.4)	0.572
Gastrointestinal	22 (6.9)	17 (7.4)	
Hepatobiliary	8 (2.5)	6 (2.6)	
Central nervous	7 (2.2)	5 (2.2)	
Cardiovascular	6 (1.9)	5 (2.2)	
Skin and soft tissue	4 (1.3)	3 (1.3)	
Other sites	11 (3.5)	8 (3.5)	
Unknown	8 (2.5)	6 (2.6)	
SOFA score, mean ± SEM	7.8 ± 0.4	8.0 ± 0.4	0.425
Septic shock, no. (%)	135 (42.6%)	108 (46.8%)	0.232
Disposition in ED, no (%)			
Intensive care unit admission	107 (33.8)	75 (32.5)	0.417
General ward admission	116 (36.6)	83 (35.9)	
Death in ED	15 (4.7)	11 (4.8)	
Others	79 (24.9)	62 (26.8)	
Length of stay (days), median (IQR)	9 (4-13)	10 (4-18)	0.436
Length of antibiotics (days), median	8 (3-13)	9 (4-16)	0.361
Compliance with recommendations, no. (%)	85 (26.8)	122 (52.8)	<0.001
Broad-spectrum antibiotics ≤3 hours	185 (58.4)	160 (69.3)	0.02
Blood culture before antibiotics	247 (77.9)	198 (85.7)	0.04
Appropriate fluid resuscitation	215 (67.8)	167 (72.3)	0.206
Lactate measurement ≥2 within 6 hours	116 (36.6)	205 (88.7)	<0.001
Time to 1st antibiotics (min), median (IQR)	121 (72-188)	112 (68-180)	0.349
7-day mortality, no (%)	44 (13.9)	30 (13.0)	0.315
14-day mortality, no (%)	87 (27.4)	62 (26.8)	0.412
28-day mortality, no (%)	118 (37.2)	74 (32.0)	0.08

**Fig. 1 (abstract P13).**
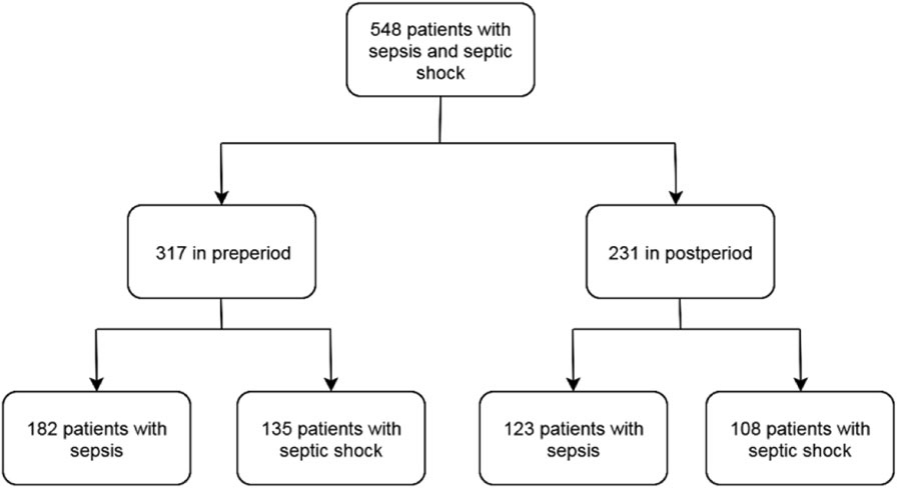
Flow chart of study population

**Fig. 2 (abstract P13).**
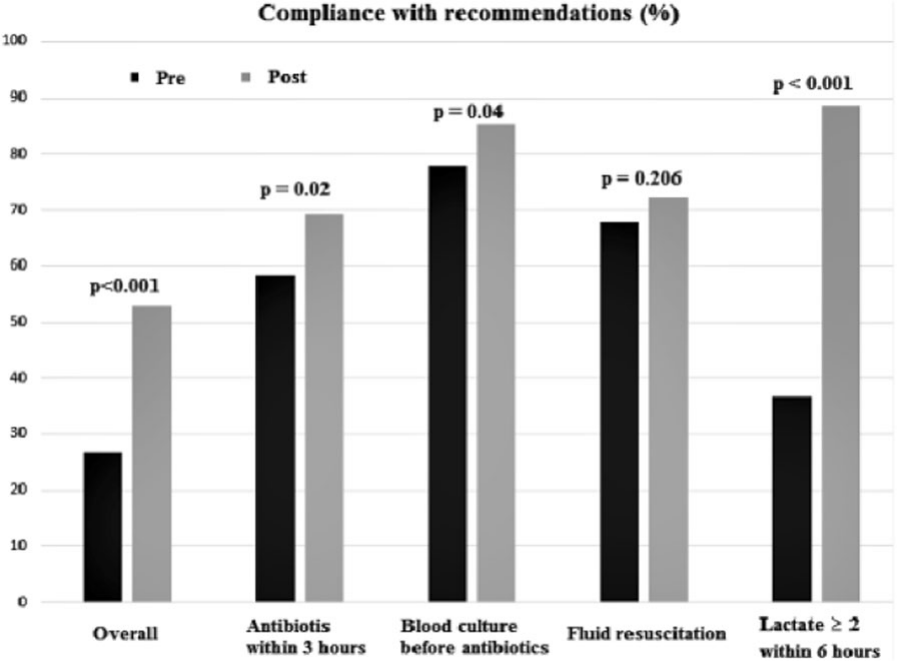
Compliance with recommendations before and after implementation of intelligent sepsis management system among patients with sepsis and septic shock

**Fig. 3 (abstract P13).**
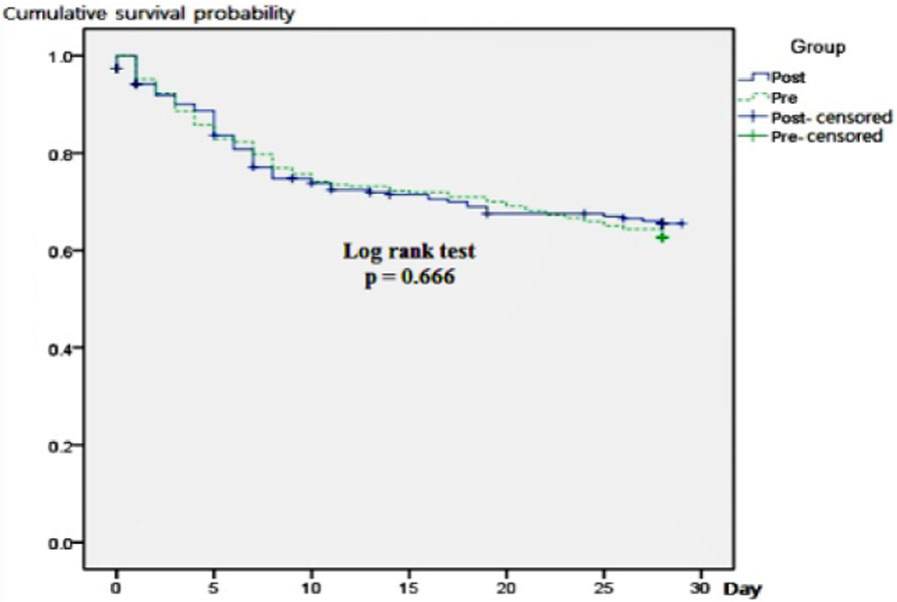
Kaplan-Meier curve of 28-day mortality before and after implementation of intelligent sepsis management system in patients with sepsis and septic Shock

**Table 2 (abstract P13).** Univariate and Multivariate Cox Regression Analysis of Risk factors for 28-Day Mortality in patients with sepsis and septic shock

	HR (95% CI)	p value	Adjusted HR (95% CI)	p value
Age	1.016 (1.006-1.028)	0.003	1.012 (1.000-1.024)	0.052
Male sex	1.002 (0.999-1.004)	0.12		
SOFA score	1.282 (1.228-1.339)	<0.001	1.265 (1.199-1.334)	<0.001
Procalcitonin	1.004 (1.000-1.009)	0.079	1.000 (0.995-1.005)	0.954
Lactate	1.080 (1.053-1.108)	<0.001	1.072 (1.032-1.112)	0.001
CRP	1.011 (0.998-1.025)	0.10	1.002 (0.985-1.019)	0.846
Time to 1^st^ antibiotics	0.999 (0.998-0.999)	0.029	0.999 (0.998-1.000)	0.050
Septic shock	2.657 (1.327-5.317)	0.004	1.247 (0.817-1.903)	0.306
Postperiod	0.939 (0.702-1.255)	0.669		

### P14 Diagnostic and prognostic value of interleukin-6, pentraxin-3, and procalcitonin levels among patients with sepsis and septic shock diagnosed at emergency department according to Sepsis-3 definitions

#### Juhyun Song^1^, Daewon Park^2^, Sungwoo Moon^1^, Hyeri Seok^2^, Sejoong Ahn^1^, Wonseok Choi^2^

##### ^1^Emergency Department, Korea University Ansan Hospital, Republic of Korea; ^2^Infectious Diseases, Korea University Ansan Hospital, Republic of Korea

###### **Correspondence:** Juhyun Song (songcap97@hotmail.com)

Background

Sepsis is a global public health problem. Despite the advances in modern medicine, more than 5.3 million people die from sepsis annually, with an estimated overall mortality of about 30% [1-3]. Early diagnosis and appropriate treatment can improve survival outcome in patients with sepsis [4]. Despite pre-existing diagnostic criteria for sepsis, early diagnosis is usually challenging due to unknown source of infection and the vague definitions of sepsis syndrome [5]. C-reactive protein (CRP) and procalcitonin (PCT) have been widely used to facilitate the diagnosis of sepsis, but the clinical values of these are limited [6, 7]. A recent study reported that IL-6 level is a diagnostic marker of infection and a prognostic marker, in patients with organ dysfunction [8]. Another study showed that PTX-3 discriminates sepsis and septic shock patients from healthy controls in a medical intensive care unit (ICU) setting [9]. However, evidence concerning the clinical value of IL-6 and PTX-3 has been controversial in several studies. The purpose of the present study was to investigate both the diagnostic and prognostic value of IL-6, PTX-3, and PCT among patients with sepsis and septic shock diagnosed at an emergency department (ED) using the latest Sepsis-3 definitions.

Materials and Methods

This study investigated biomarkers’ clinical value among patients with sepsis and septic shock. Among 143 enrolled subjects (51 with sepsis, 46 with septic shock, and 46 healthy volunteers), serum levels of IL-6, PTX-3, and PCT were measured (Table 1). Follow-up IL-6 and PTX-3 levels were measured among patients with initial septic shock within 24 hours of hospital discharge. Optimal cut-off values were obtained for sepsis and septic shock. Prognostic value was evaluated by Cox regression analysis and Kaplan-Meier survival analysis.

Results

The median values (IQR) of IL-6 in controls, sepsis, and septic shock were 0.6 (0.2-1.6), 89.9 (45.2-272.6), and 1378.6 (256.4-11062.1) pg/ml, respectively (Figure 1). Serum IL-6 levels could discriminate sepsis (AUC, 0.97-1.00, p<0.001; cut-off value, 5.89 pg/mL [sensitivity 97.0%, specificity 97.2%]) from controls and could discriminate septic shock (AUC, 0.85-0.95; cut-off value, 53.59 pg/mL [sensitivity 91.8%, specificity 63.2%]) from the others (controls and sepsis) (Table 2 and Figure 2). Twenty-eight-day mortality was significantly higher in the high IL-6 (≥53.59 pg/ml) group than in the low IL-6 (<53.59 ng/ml) group (p=0.002) (Figure 3). IL-6 was an independent risk factor for 28-day mortality among patients with sepsis and septic shock (HR, 1.0004; 95% CI, 1.0003-1.0005; p=0.024) (Table 3). PTX-3 level was not significant in the multivariate Cox regression analysis (HR, 1.003; 95% CI, 0.998-1.008; p=0.095) (Table 3). Both initial and follow-up PTX-3 levels of septic shock patients who died during admission were consistently significantly higher than those of septic shock patients who recovered (initial: p=0.004, follow-up: p<0.001) (Figure 4).

Conclusions

IL-6 level was superior to PTX-3 and PCT levels in both diagnostic and prognostic value for sepsis and septic shock diagnosed in the emergency department using Sepsis-3 definitions. IL-6 level was an independent risk factor for 28-day mortality.

Acknowledgements

The authors thank research nurse Hye-Yoon Jung and researcher Min-Sook Jung for their contributions to the project. We also thank Jae-Hyung Cha, PhD for lending statistical support.

References

1. Singer M, Deutschman CS, Seymour CW, Shankar-Hari M, Annane D, Bauer M, Bellomo R, Bernard GR, Chiche J-D, Coopersmith CM, et al. The Third International Consensus Definitions for Sepsis and Septic Shock (Sepsis-3). JAMA. 2016; 315(8):801-10.

2. Rhodes A, Evans LE, Alhazzani W, Levy MM, Antonelli M, Ferrer R, Kumar A, Sevransky JE, Sprung CL, Nunnally ME, et al. Surviving Sepsis Campaign: International Guidelines for Management of Sepsis and Septic Shock: 2016. Crit Care Med. 2017; 45(3):486-552.

3. Angus DC, van der Poll T. Severe sepsis and septic shock. N Engl J Med. 2013; 369(9):840-51.

4. De Backer D, Dorman T. Surviving Sepsis Guidelines: A Continuous Move Toward Better Care of Patients With Sepsis. JAMA. 2017; 317(8):807-8.

5. Biron BM, Ayala A, Lomas-Neira JL. Biomarkers for Sepsis: What Is and What Might Be? Biomark Insights. 2015; 10(Suppl 4):7-17.

6. Kibe S, Adams K, Barlow G. Diagnostic and prognostic biomarkers of sepsis in critical care. J Antimicrob Chemother. 2011; 66 Suppl 2:ii33-40.

7. Henriquez-Camacho C, Losa J. Biomarkers for sepsis. Biomed Res Int. 2014; 2014:547818.

8. Takahashi W, Nakada TA, Yazaki M, Oda S. Interleukin-6 Levels Act as a Diagnostic Marker for Infection and a Prognostic Marker in Patients with Organ Dysfunction in Intensive Care Units. Shock. 2016; 46(3):254-60.

9. Hamed S, Behnes M, Pauly D, Lepiorz D, Barre M, Becher T. Diagnostic value of Pentraxin-3 in patients with sepsis and septic shock in accordance with latest sepsis-3 definitions. BMC Infect Dis. 2017; 17(1):554.

**Table 1 (abstract P14).** Baseline Characteristics of Study Population

	Total (n = 97)	Sepsis (n = 51)	Septic shock (n = 46)	Controls (n = 46)
Age, median (range)	75 (42-98)	76 (42-98)	74 (42-96)	66 (35-79)
Sex, n (%)				
Male	54 (56)	28 (55)	26 (57)	24 (52)
Female	43 (44)	23 (45)	20 (43)	22 (48)
Infection site, n (%)				
Respiratory	63 (64.9)	33 (64.7)	30 (65.2)	
Genitourinary	32 (33.0)	19 (37.3)	13 (28.3)	
Cardiovascular	3 (3.1)	0 (0.0)	3 (6.5)	
Gastrointestinal	2 (2.1)	0 (0.0)	2 (4.3)	
Musculoskeletal	2 (2.1)	1 (2.0)	1 (2.2)	
Central Nervous	1 (1.0)	0 (0.0)	1 (2.2)	
Hepatobiliary	1 (1.0)	0 (0.0)	1 (2.2)	
Skin and soft tissue	1 (1.0)	1 (2.0)	0 (0.0)	
Unknown	5 (5.2)	2 (3.9)	3 (6.5)	
Underlying disease				
Coronary artery disease	11 (11.3)	5 (9.8)	6 (13.0)	
Malignancy	10 (10.3)	4 (7.8)	6 (13.0)	
Rheumatic disease	4 (4.1)	2 (3.9)	2 (4.3)	
SOFA score, median (IQR)	8 (4-11)	6 (3-9)	10 (6-13)	
APACHE II score, median (IQR)	21 (13-30)	18 (10-27)	25 (16-35)	
Laboratory value, median (IQR) or mean ± SEM				
Procalcitonin (ng/ml)	1.6 (0.5-10.7)	0.3 (0.2-1.2)	3.4 (1.6-20.3)	
CRP (mg/dL)	10 (6-20)	10 (5-20)	11 (7-21)	
Lactate (mmol/L)	3.6 (2.6-4.6)	1.9 (1.1-2.8)	5.5 (3.6-7.5)	
Creatinine (mg/dL)	2.5 ± 0.2	2.1 ± 0.2	2.9 ± 0.2	
Bilirubin (mg/dL)	2.2 ± 0.4	1.7 ± 0.3	2.8 ± 0.5	
Platelet (×1000/μL)	203 ± 12.4	251 ± 14.3	153 ± 10.6	
Positive blood cultures, n (%)	75 (77.3)	35 (68.6)	40 (87.0)	
ICU days, median (IQR)	9 (5-14)	8 (4-11)	11 (7-16)	
Length of stay, median (IQR)	13 (8-18)	11 (7-16)	15 (9-19)	

*SOFA* sequential organ failure assessment, *IQR* interquartile range, *APACHE* acute physiology and chronic health evaluation, *CRP* C-reactive protein, *SEM* standard error of the mean, *ICU* intensive care unit

**Table 2 (abstract P14).** Diagnostic Value of Interleukin-6 (IL-6), Pentraxin-3 (PTX-3), and Procalcitonin (PCT) for Patients with Sepsis and Septic Shock in the Emergency Department

	Severity	AUC (95% CI)	Cutoff value	Sensitivity (%)	Specificity (%)	P value
IL-6	Sepsis	0.99 (0.97-1.00)	5.89	97.0	97.2	<0.001
(pg/ml)	Septic shock	0.90 (0.85-0.95)	53.59	91.8	63.2	<0.001
PTX-3	Sepsis	0.97 (0.95-0.99)	6.02	92.6	97.4	<0.001
(ng/ml)	Septic shock	0.84 (0.77-0.90)	12.05	93.2	60.7	<0.001
PCT	Sepsis	0.91 (0.86-0.96)	0.23	77.7	94.9	<0.001
(ng/ml)	Septic shock	0.86 (0.79-0.92)	0.83	75.0	83.1	<0.001

*AUC* area under the curve, *CI* confidence interval

**Table 3 (abstract P14).** Univariate and Multivariate Cox Proportional Model of Risk Factors for 28-day Mortality

	HR (95% CI)	p value	Adjusted HR (95% CI)	p value
Age	1.023 (0.994-1.052)	0.120		
Male sex	1.029 (0.535-1.978)	0.932		
GCS score	0.777 (0.692-0.873)	<0.001	0.777 (0.695-0.869)	<0.001
SOFA score	1.206 (1.076-1.353)	0.001	1.048 (0.901-1.219)	0.208
APACHE II score	1.198 (1.068-1.346)	0.001	1.031 (0.898-1.187)	0.231
Pentraxin-3	1.005 (1.001-1.009)	0.031	1.003 (0.998-1.008)	0.095
Interleukin-6	1.001 (1.000-1.002)	0.017	1.001 (1.000-1.002)	0.024
Procalcitonin	0.995 (0.981-1.009)	0.481		
Lactate	1.167 (1.068-1.275)	0.001	1.135 (1.033-1.247)	0.009
CRP	1.011 (0.978-1.045)	0.525		
Septic shock	2.657 (1.327-5.317)	0.004	1.249 (0.472-3.302)	0.240

*HR* hazard ratio, *CI* confidence interval, *GCS* Glasgow coma scale, *SOFA* sequential organ failure assessment, *APACHE* acute physiology and chronic health evaluation, *CRP* C-reactive protein

**Fig. 1 (abstract P14).**
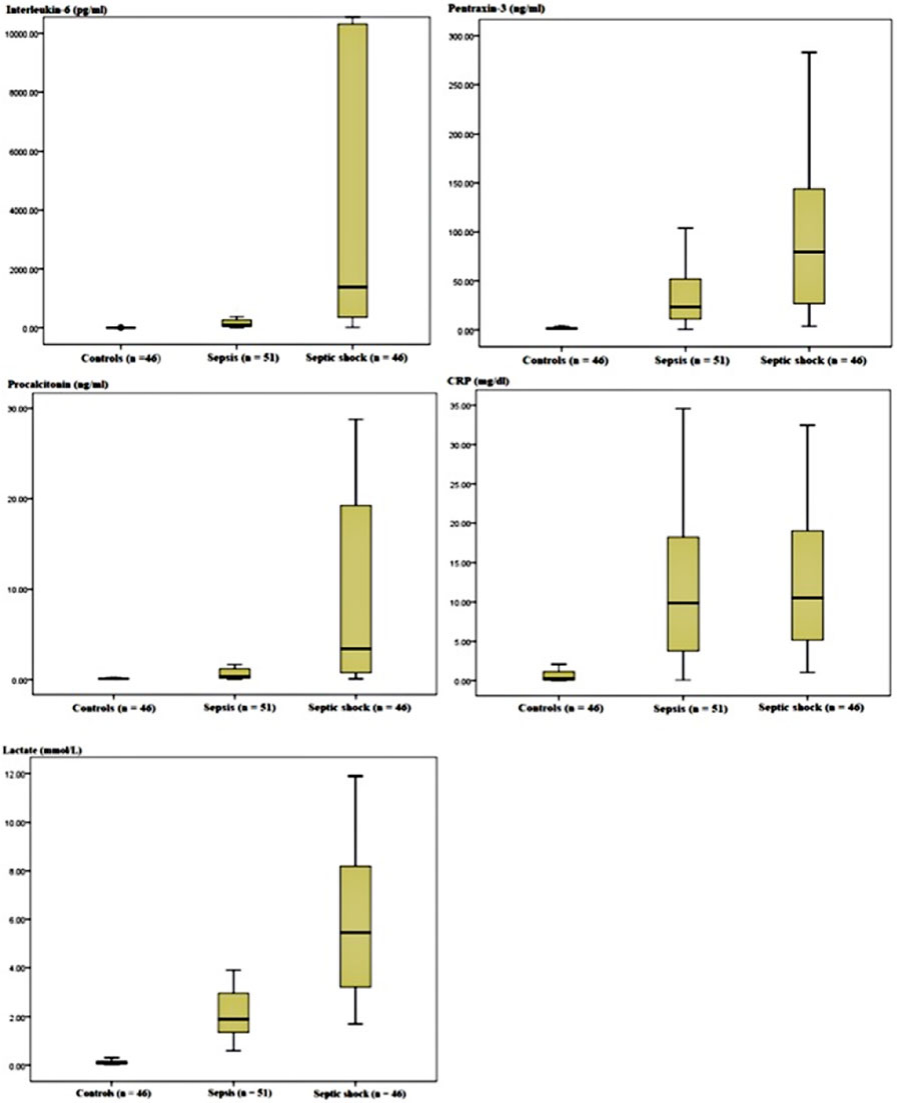
Interleukin-6, Pentraxin-3, Procalcitonin, CRP, and Lactate Levels in Patients with Sepsis and Septic Shock Diagnosed in the Emergency Department according to Sepsis-3 Definitions. CRP, C-reactive protein

**Fig. 2 (abstract P14).**
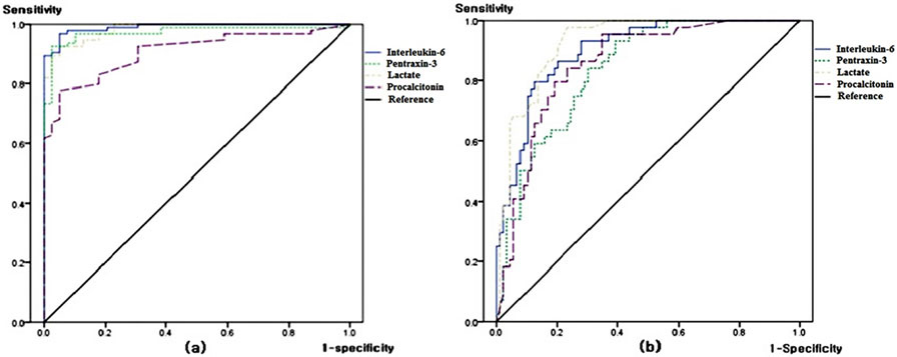
Receiver Operating Characteristic (ROC) Curves to Discriminate Sepsis (a) and Septic Shock (b) by Interleukin-6, Pentraxin-3, Lactate, and Procalcitonin Levels Measured in the Emergency Department

**Fig. 3 (abstract P14).**
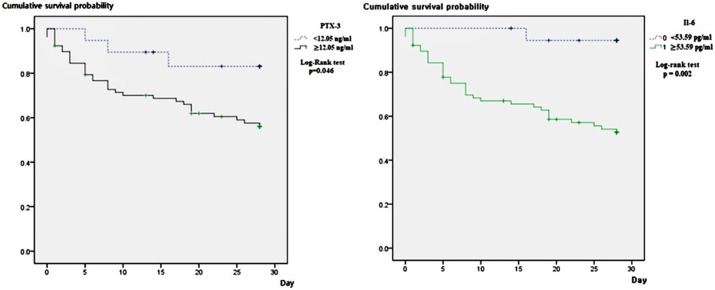
Kaplan-Meier Curve of 28-day Mortality in Patients with Sepsis and Septic Shock Stratified by the Optimal Cut-off Value of Pentraxin-3 (a) and Interleukin-6 (b) for Predicting Septic shock (28-day mortality by pentraxin-3: 16.9% vs 43.9%, 28-day mortality by interleukin-6: 5.6% vs 47.4%)

**Fig. 4 (abstract P14).**
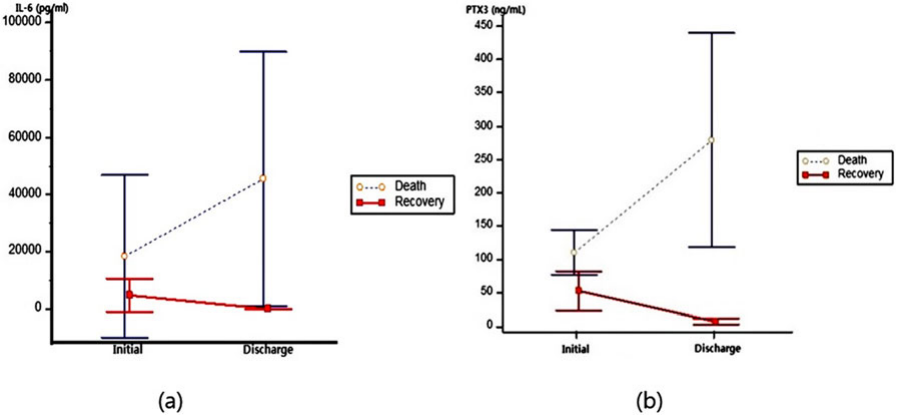
Error Bars of Initial (within 6 hours of Clinical Diagnosis) and Follow-up (within 24 hours of Discharge) Levels of Interleukin-6 (a) and Pentraxin-3 (b) in Septic Shock Patients who Died or Recovered during Admission

### P15 Combined prognostic role of interleukin-35 and presepsin (sCD14 subtype) in clinical setting of sepsis and septic shock

#### Cinzia Peronace^1^, Maria Teresa Loria^1^, Carolina Mirello^1^, Aida Giancotti^1^, Paola Morelli^1^, Nadia Marascio^1^, Eugenio Garofalo^2^, Andrea Bruni^2^, Paolo Navalesi^2^, Angela Quirino^1^, Maria Carla Liberto^1^, Giovanni Matera^1^

##### ^1^Department of Health Sciences, Operating Unit of Microbiology, “Magna Graecia” University, Catanzaro, Italy; ^2^Operating Unit of Intensive Care, “Magna Graecia” University, Catanzaro, Italy

###### **Correspondence:** Giovanni Matera (gm4106@gmail.com)

Background

The detection of sepsis-specific biomarkers ameliorate recognition and management of sepsis by improvement: diagnosis, monitoring response to treatment, and stratifying patients based on prognosis or underlying biological response. The diagnostic and prognostic role of novel biomarkers of sepsis is the aim of our study. In particular, we have investigated the kinetic of IL-35, presepsin and procalcitonin in critical septic patients with poor prognosis.

Materials and Methods

Fifty-nine critical septic patients admitted to ICU of the University Hospital of Catanzaro (Italy) were enrolled; a group of healthy volunteers were also included. All studied subjects were stratified into survivors and nonsurvivors, based on 28 days survival and according to microbiological results in blood culture positive and blood culture negative groups. Clinical data and blood samples were collected for quantitative analysis of IL-35, sCD14-ST and PCT at: i) time 0 (T0): time at which the first blood culture for analysis was collected; ii) time T minus 7 (T-7): 7 days before exitus or hospital discharge; iii) time T minus 2 (T-2): 2 days before exitus or hospital discharge. Data were subjected to statistical analysis by ANOVA plus PLSD test. A p value < 0.05 was considered statistically significant.

Results

Interleukin-35 levels were found significantly (p< 0.05) increased in the group of eventually dead patients, both at T0 and at T-2 times. By contrary, such different behavior between alive and dead patients did not achieve any significant difference at T-7; although the group of patients with a poor prognosis still exhibited a light increase of IL-35. RegardingsCD14-ST, our data confirmed that presepsin is a valuable prognostic biomarker for the studied patients (p< 0.05). PCT exhibited a significantly lower levels in the eventually non-survivors only at time T0; by contrary both at times T-7 and T-2 PCT levels of dead patients were found significantly higher than survivors.

Conclusions

Serial increase of IL-35 and sCD14-ST or presepsin reveals prognostic value for in hospital mortality compared to PCT as observed at T0. Recent study shown the intrisic multimodal co-existing pro-and anti-inflammatory mechanisms reflect specific immune reprogramming [1].

Reference

1. Denstaedt SJ, Singer BH, Standiford TJ. Sepsis and Nosocomial Infection:Patient Characteristics, Mechanisms, and Modulation. Front Immunol. 2018 9:2446. doi: 10.3389/fimmu.2018.02446

### P16 Rapid identification of *Listeria monocytogenes* from vertebral joint abscess clinical sample using eSensor and Electrowetting technology

#### Angela Quirino^1^, Gallo Luigia^1^, Francesca Divenuto^1^, Maria Concetta Reale^1^, Cinzia Peronace^1^, Giorgio Settimo Barreca^1^, Angelo Giuseppe Lamberti^1^, Enrico Maria Trecarichi^2^, Carlo Torti^2^, Giorgio Gasparini^3^, Maria Carla Liberto^1^, Giovanni Matera^1^

##### ^1^Department of Health Sciences, Operating Unit of Microbiology, “Magna Graecia” University, Catanzaro, Italy; ^2^Operating Unit of Infectious and Tropical Diseases, “Magna Graecia University”, Catanzaro, Italy; ^3^Operating Unit of Orthopaedic and Trauma Surgery, “Magna Graecia University”, Catanzaro, Italy

###### **Correspondence:** Giovanni Matera (gm4106@gmail.com)

Background

*Listeria monocytogenes (L. monocytogenes)* is a facultative intracellular Gram-positive bacterium that infects humans through food. The broad clinical spectrum of Listeria monocytogenes infections includes frequent clinical forms, such as meningitis or bacteremia, and uncommon manifestations, such as septic arthritis. Osteoarticular infections due to *L. monocytogenes* have been reported very rare. We report on a case of osteoarticular abscess, which developed into a bacteremia both caused by *L. monocytogenes* in an older adult, using a rapid identification by innovative technology.

Materials and Methods

An 83-year-old man was admitted to our university hospital with a retroperitoneal abscess and acute low back pain suggestive of spondylodiscitis. He had no sign of meningoencephalitis or fever. A clinical sample from vertebral joint abscess and some blood cultures were sent to our laboratory for microbiological diagnosis. Sepsis biomarkers (procalcitonin and sCD14-ST) were also required. All of the samples were analyzed by conventional methods. Moreover, vertebral joint abscess was analyzed by ePlex device, that identifies a broad panel of pathogens and resistance genes, using BCID-GP Panel (GenMarkDx, USA). The system relies on electrowetting technology to perform multiplexed nucleic acid extraction, amplification and digestion followed by the detection of analyte targets using eSensor technology. ePlex device, has been approved and commercialized for positive blood cultures only. We used this molecular technology directly on the sample of vertebral joint abscess. Positive blood cultures were assessed by molecular method using the FilmArray system (BioMerieux, Italy). Serum procalcitonin and presepsin were tested by ELFA and CLEIA methods respectively.

Results

ePlex system identified *L. monocytogenes* in about 90 minutes directly on sample of vertebral joint abscess. Moreover, *L. monocytogenes* was identified using the FilmArray blood culture ID Panel in about one hour on positive blood cultures. Sub-cultures, processed using conventional and proteomic methods, confirmed molecular diagnosis results.

Conclusions

Rapid detection of *L. monocytogenes* by innovative technology allowed the clinicians to establish an early antibiotic therapy. This, as well as other molecular techniques, may represents an integrated approach to conventional methods for etiological diagnosis.

### P17 After recovery from severe-sepsis, the lung carries significant quiescent lesions with potential recurrent pathogenicity

#### Rodrigo B Souza, Ana MA Liberatore, Ivan HJ Koh

##### Department of Surgery, Escola Paulista de Medicina, Universidade Federal de São Paulo, São Paulo, Brasil

###### **Correspondence:** Ivan HJ Koh (ivankoh@terra.com.br)

Background

The causal role of sepsis on the long-term impairment and survival remains unclear, but is a fact that survivors of sepsis are profoundly functionally impaired. The lung is the organ often affected in sepsis and course with high lethality by ARDS. Thus, this study aimed to evaluate the pulmonary status in survivors of a severe sepsis in search of remaining lesions with potential recurrence of pulmonary disease.

Materials and Methods

Under general anesthesia, Wistar rats (250g) were submitted to severe sepsis (iv. inoculation of 2 mL *Escherichia coli* 108 CFU/ mL), that course with hypotension within 4-6 hours and with 50-60% mortality within 26 hours. Lung of the survivals was collected at 30 and 90 days under general anesthesia and were sacrificed after. (n=4/period). The samples were submitted to histological study using HE dye and results were compared to lung of naïve (T0) and septic animals of 6hours after challenge (n=4/period).

Results

The 6h sepsis group showed a vascular congestion of the alveolar wall, alveolar wall thickening and neutrophils infiltration, and BALT hyperplasia, in a focal manner. (Figure 1). After 30 days of sepsis was observed multifocal thickening of alveoli walls with intense and broad congestion of capillaries. The BALT hyperplasia also was more intense as compared to 6h findings. These results showed a continuing inflammatory process and suggested that a state of an acute inflammatory reaction coexists with apparent clinical normality. In 90 days after sepsis induction, although all animals remained clinically asymptomatic, significant pulmonary lesions were seen, with intense capillaries congestion, broad atelectasis, severe thickening of the alveolar wall, infiltrate of mono and polynuclear cells in most of alveoli, multiple areas with hemorrhage, peri-bronchial leukocyte infiltrates, atherosclerosis, and intense BALT hyperplasia associated to obstruction of bronchioles and multiple areas of atelectasis. These findings demonstrated that healthy-looking animals were concealing a functionally impaired lung structure combined to an intense inflammatory reaction state in course, denoting an impaired lung physiological condition to face with new deleterious stimuli in sepsis survivors.

Conclusions

A severely compromised state of lung following sepsis recovery might explain the ease for higher susceptibility for recurrent pulmonary diseases. These events are possibly related to the post-sepsis syndrome that courses with a persistent immunosuppression, inflammation and catabolism. Clinical studies are needed to confirm these animal experimental findings. In the future, understanding of mechanisms supporting these persistent lung injuries may afford intervention targets to ameliorate post-sepsis high mortality.

Acknowledgements

Grant acknowledgment: FAPESP 2017/21052-0

References

1. Won-Young Kim and Sang-Bum Hong: Sepsis and Acute Respiratory Distress Syndrome: Recent Update. Tuberc Respir Dis 2016;79:53-57.

2. Manu Shankar-Hari & Gordon D. Rubenfeld: Understanding Long-Term Outcomes Following Sepsis: Implications and Challenges. Curr Infect Dis Rep (2016) 18: 37.

3. Sorin Hostiuc, Dan Dermengiu, Mihai Ceaușu, Mugurel Constantin Rusu, George Cristian Curcă: Pathology and immunopathology of the lung in sepsis. Rom J Leg Med [19] 83-88 [2011].

**Fig. 1 (abstract P17).**
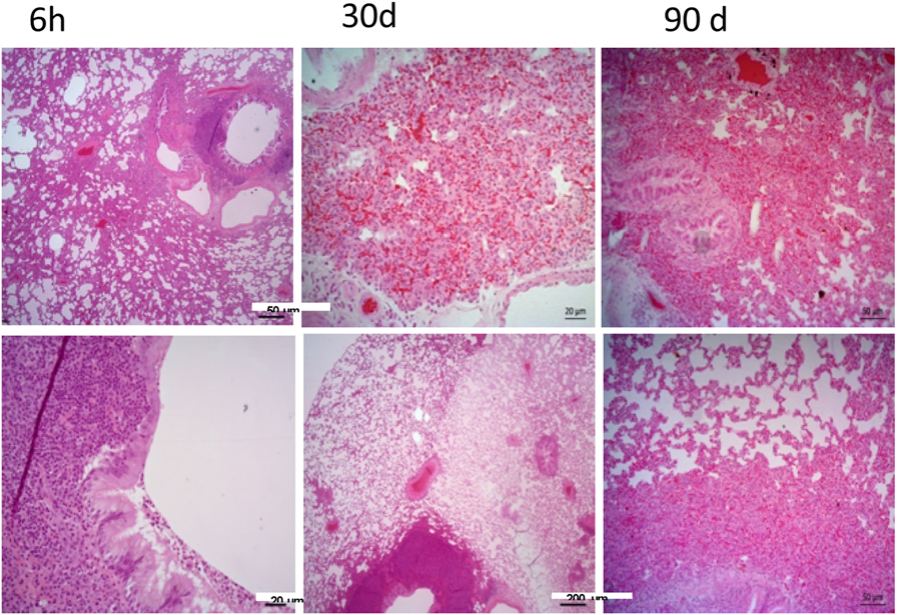
Pathological aspects found in the lung of sepsis survivors in the periods of 6 hours, 30 days and 60 days after induction of severe sepsis

### P18 Can we do it better together? A Derbyshire countywide approach to sepsis care

#### Alina M. Paunescu^1^, Duncan Cameron^2^

##### ^1^Emergency Medicine Consultant, University Hospitals of Derby and Burton NHS Foundation Trust, Derby, United Kingdom; ^2^Transformation Team Lead, University Hospitals of Derby and Burton NHS Foundation Trust, Derby, United Kingdom

###### **Correspondence:** Alina M. Paunescu (alina.paunescu@nhs.net)

Background

In the United Kingdom 70% of sepsis cases are derived from an infection developed in the community[1]. It is estimated that there is potential to reduce deaths by up to 10,000 per year by the optimization of care.

Mortality review of sepsis deaths in Royal Derby Hospital for November 2017 revealed that 50% of patients died in the first couple of hours to 1 day following admission. Of these, 85% were previously treated in the community with antibiotics for different periods of time, some of them with repeated visits to their General Practitioner

The findings of the reviews suggested communication deficits and inconsistent pathways between primary and secondary care.

Materials and Methods

A Derbyshire wide project group was set up at the authors suggestion, including commissioners, the ambulance service, out of hours service, community providers, primary and secondary care. The final aim of the project was improving sepsis outcomes by adopting a standard approach to screening and treatment countywide, at all levels of care. We have conducted process mapping of the pathways and procedures each provider followed when dealing with a potential sepsis case and identified where delays/ inconsistencies/ issues existed.

The agreed action plan:

- Adopt the same escalation triggers and similar screening methods
- Reduce the time from identification to treatment especially in remote rural areas of Peak District
- Increase awareness/education of Sepsis and Derbyshire pathways across providers
- Improve sepsis treatment, review antibiotic protocols and patient education about antibiotics
- Develop post sepsis support

Results

Important steps have been achieved following our intervention (Table 1) starting with agreement on using same new early warning scores (NEWS 2) and paediatric observation priority score (POPS) throughout Derbyshire. It is now possible for sepsis treatment to be initiated in the community long before the patient reaches hospital through education of community nurses and general practitioners’ agreement. While the initiative is ongoing due to the massive ramifications of the project, intermediate data showed that crude mortality from sepsis is on a continuous downward trend (Graph 1).

Conclusions

Developing a unified approach and increasing communication between providers generated benefits for patient, practitioners and local health economy. While raw data analysis makes it easy to measure patient outcomes, the benefits of creating a local peer learning resource and support network are not easily quantifiable and can only be predicted as positive.

Acknowledgements

1. United Kingdom, Derbyshire Sepsis Group, United Kingdom

2. Joanna Harisson, Urgent Care Analyst, University Hospitals of Derby and Burton NHS Foundation Trust, Derby,

Reference

1. Donald M Yealy, David T Huang Anthony Delaney, Marian Knight, Adrienne G Randolph, Ron Daniels and Tim Nutbeam, Recognizing and managing sepsis :what needs to be done?, BMC Med.2015;13:98, Published online 2015 Apr 27, PMCID:PMC4410741, PMID:25927426

**Fig. 1 (abstract P18).**
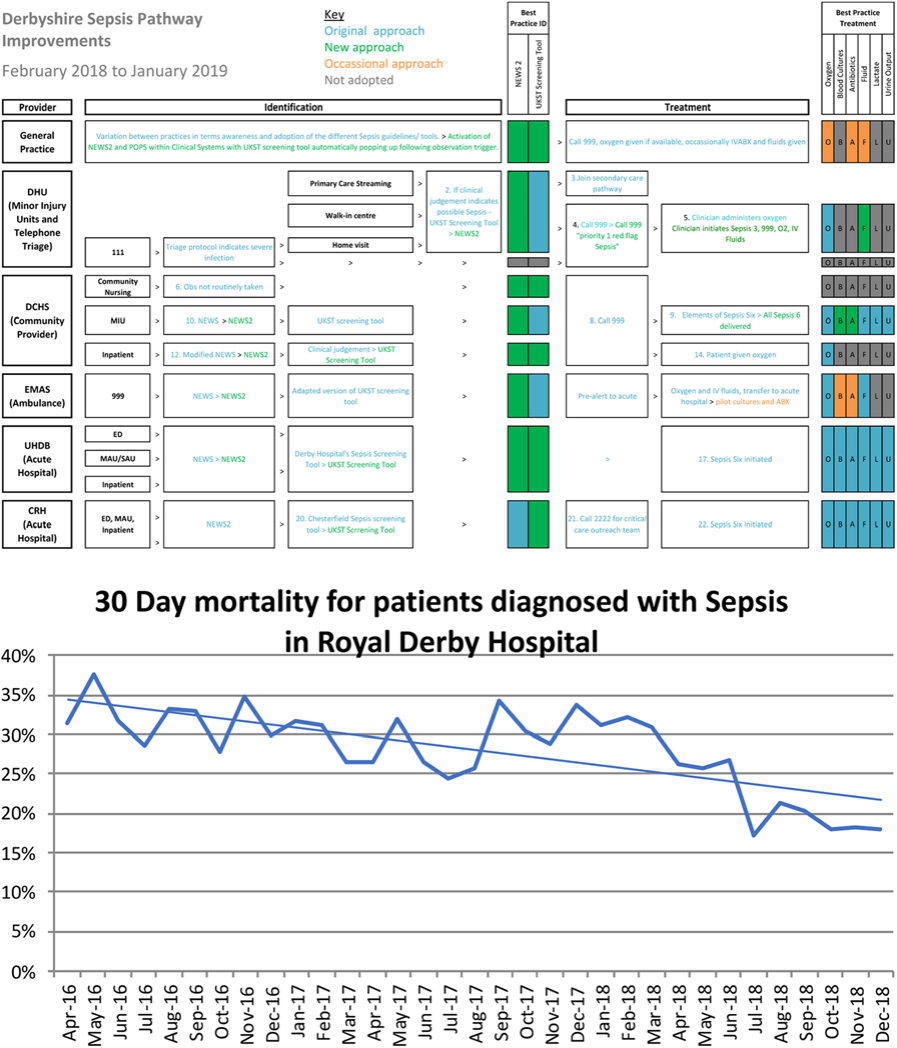
See text for description

### P19 Targeting the NO-sGC axis to monitor and treat vascular dysfunction and vasoplegia in sepsis

#### Forough Jahandideh^1^, Sareh Panahi^1^, Kimberley Macala^1^, Stephane L. Bourque^1,2^

##### ^1^Department of Anesthesiology and Pain Medicine, University of Alberta, Edmonton, Canada; ^2^Department of Physiology, University of Alberta, Edmonton, Canada

###### **Correspondence:** Stephane L. Bourque (sbourque@ualberta.ca)

Background

Sepsis is a life-threatening condition caused by a dysregulated host response to infection. If unimpeded, sepsis can progress to septic shock, characterized by refractory hypotension and unresponsive vasculature (i.e. vasoplegia) resulting in tissue hypoperfusion and eventually organ failure [1]. The overall mortality rate of septic shock is more than 50%. The vascular dysfunction and refractory hypotension in septic shock are caused, at least in part, by excess reactive oxygen species (ROS) generation and dysregulated nitric oxide (NO) production (via inducible NO synthase upregulation and increased scavenging by excess ROS) [2,3,4]. Moreover, excess ROS oxidizes and hence damages soluble guanylate cyclase (sGC), the downstream mediator of NO, resulting in the impaired organ blood flow [5,6]. We believe the loss of sGC-mediated vasodilation is a critical determinant of reduced organ blood flow in sepsis. The objective of this study is to assess the state of vascular dysfunction and altered blood flow patterns as sepsis progresses to septic shock. We also sought to assess the efficacy of (i) the sGC activator cinaciguat or (ii) the superoxide dismutase mimetic tempol in improving hemodynamics and survival in a murine model of sepsis.

Materials and Methods

The experimental protocols described herein have been approved by the University of Alberta Animal Care and Use Committee in conformance with the FASEB Statement of Principles for the use of Animals in Research and Education. Male C57Bl/6 mice were instrumented with fiber-optic pressure sensors for direct blood pressure monitoring. Flow probes were placed around the left common carotid, superior mesenteric, and right renal arteries to monitor blood flow to the brain, gut, and kidney, respectively. After baseline recordings, sepsis was induced by an intraperitoneal injection of a fecal slurry. Thirty minutes after induction of sepsis, mice were treated with the cinaciguat (15μg/kg IV) or tempol (30mg/kg, IV).

Results

The blood pressure in septic mice reduced significantly over time (44% ± 4% reduction after 4 h of fecal slurry injection) compared to control mice (Figure 1A), but no significant changes in heart rate were noted (Figure 1B). Blood flow in the carotid, superior mesenteric, and renal arteries reduced at 47 ± 4%, 71 ± 8%, and 57 ± 13% respectively, 4 hours after fecal slurry injection (Figures 2A, B, and C, respectively). Vessel reactivity, assessed by monitoring constrictor and relaxation responses to bolus doses of phenylephrine (10 μg/kg body weight) and sodium nitroprusside (5 μg/kg body weight) was reduced by 36 ± 9% and 53 ± 7% respectively, in septic mice compared to controls, suggesting impaired regional vascular function. These data suggest certain organs are more susceptible to vascular dysfunction and hypoperfusion with the progression of sepsis. While a low dose (15μg/kg IV) of cinaciguat provided the best survival outcomes in our model, our preliminary data have also shown that administration of tempol (30mg/kg, IV), 30min after injection of fecal slurry, prolongs survival and mitigates the decline in blood pressure and superior mesenteric artery blood flow.

Conclusions

The proposed therapeutic is expected to reduce organ damage and improve blood flow to organs without causing systemic vasodilation and hypotension in sepsis.

References

1. Singer M, Deutschman CS, Seymour CW, Shankar-Hari M, Annane D, Bauer M, Bellomo R, Bernard GR, Chiche JD, Coopersmith CM, Hotchkiss RS, Levy MM, Marshall JC, Martin GS, Opal SM, Rubenfeld GD, van der Poll T, Vincent JL, Angus DC. The third international consensus definitions for sepsis and septic shock (sepsis-3). JAMA. 2016;315:801-810.

2. Kimmoun A, Ducrocq N, Levy B. Mechanisms of vascular hyporesponsiveness in septic shock. Curr Vasc Pharmacol. 2013;11:139-149.

3. Muhl H, Bachmann M, Pfeilschifter J. Inducible NO synthase and antibacterial host defense in times of Th17/Th22/T22 immunity. Cell Microbiol. 2011;13:340-348.

4. Wong CM, Au CL, Tsang SY, Lau CW, Yao X, Cai Z, Chung AC. Role of inducible nitric oxide synthase in endothelium-independent relaxation to raloxifene in rat aorta. Br J Pharmacol. 2017;174:718-733.

5. Gutterman DD, Chabowski DS, Kadlec AO, Durand MJ, Freed JK, Ait-Aissa K, Beyer AM. The human microcirculation: Regulation of flow and beyond. Circ Res. 2016;118:157-172.

6. Bateman RM, Sharpe MD, Ellis CG. Bench-to-bedside review: Microvascular dysfunction in sepsis--hemodynamics, oxygen transport, and nitric oxide. Crit Care. 2003;7:359-373.

**Fig. 1 (abstract P19).**
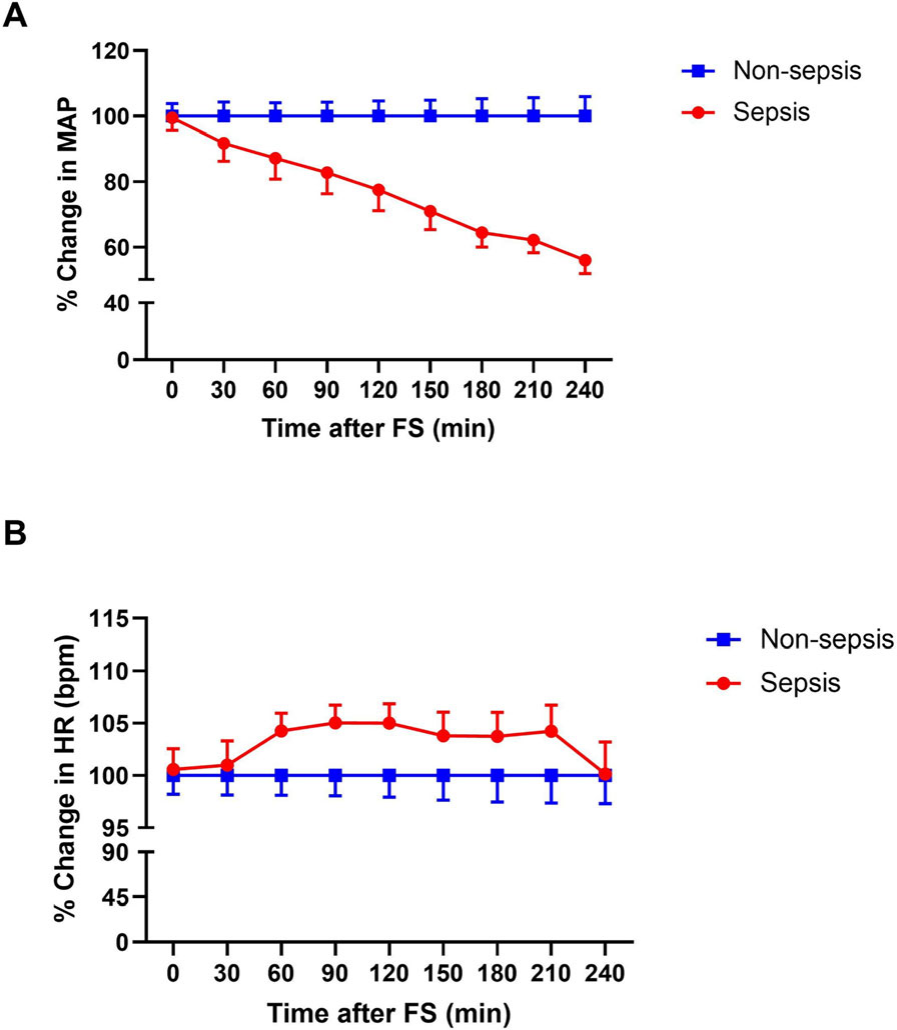
See text for description

**Fig. 2 (abstract P19).**
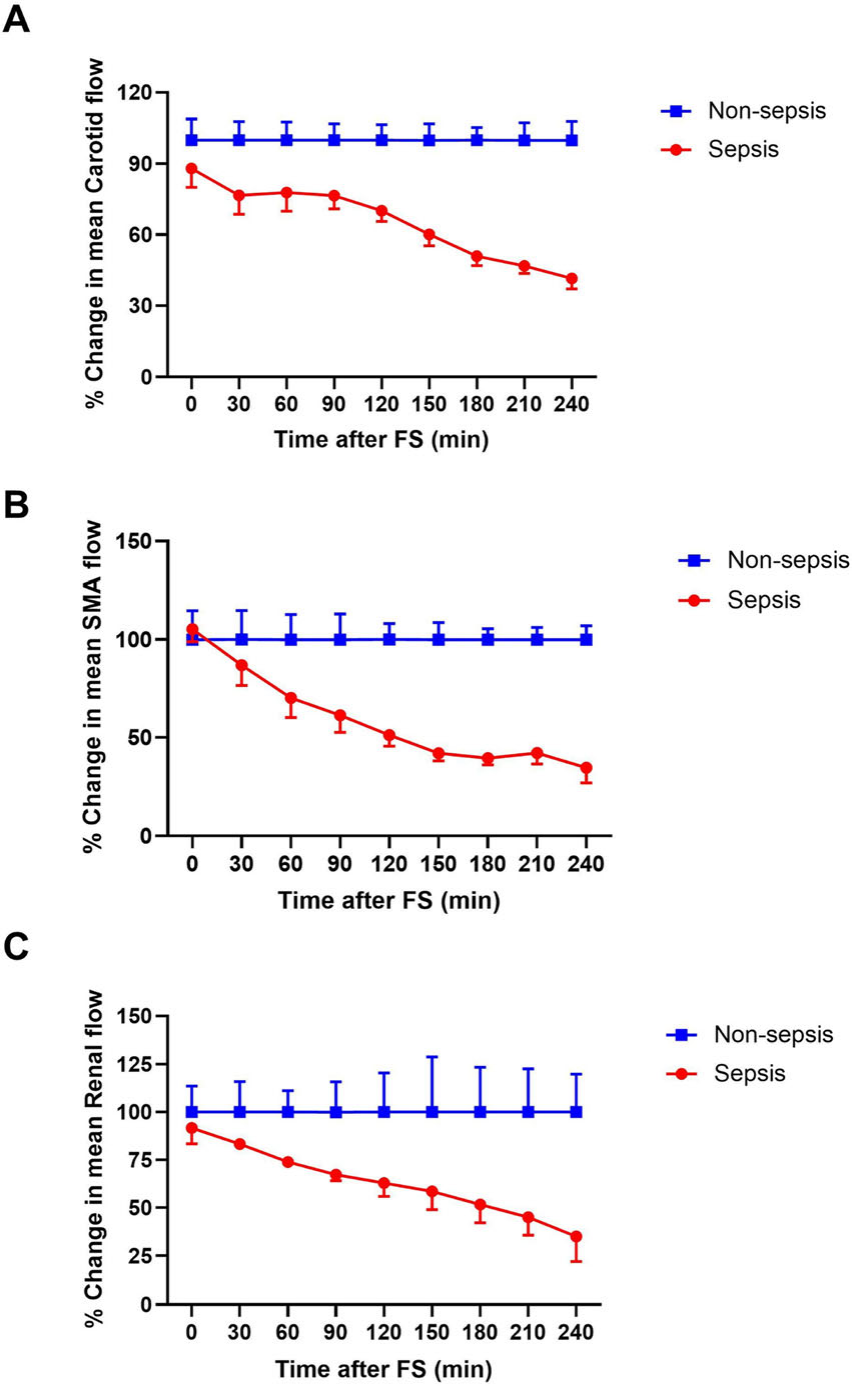
See text for description

### P20 Primary peritonitis with rapid evolution to abdominal sepsis in an immunocompetent patient

#### Daniel N. de Almeida¹, Vitória S. Freitas¹, Pamela M. Ureta²

##### ^1^Centro Universitário Serra dos Órgãos, UNIFESO, Teresópolis, Brazil; ^2^Universidad Nacional de la Matanza, UNLAM, Buenos Aires - Argentina

###### **Correspondence:** Daniel N. de Almeida (daniel_nalmeida@hotmail.com)

Background

We present the case of a 55-year-old Argentinian woman, with a clinical of peritonitis with 24h of evolution to sepsis, characterized initially by acute diffuse abdominal pain associated with diarrhea without mucus or blood. Patient denied nausea, evolving to abdominal distention, absence of peristalsis and signs of peritoneal irritation, negative catharsis and absence of flatulence with hemodynamic decompensation.

Case Report

In the physical examination, she was oriented in time and space, cooperative, Glasgow 15/15, no fever (36ºC), hypotensive (80/60mmHg), tachycardic 112 bpm, with respiratory rate at 18 breaths per minute, abdomen distended, painful at superficial palpation and deep peritoneal resistance, absence of intestinal sounds, symmetric extremities and venous return lower than 2 seconds. The following complementary exams were made: electrocardiogram and chest X-Ray, with the imaging of the X-Ray being interpreted as septic shock and validated by an intensive care unit professional[1].

During the 10 weeks hospitalized, the patient underwent various procedures following the diagnosis, including reanimation with vasopressors and this medication caused ischemia and necrosis of phalanges later, exploratory laparotomy, bilateral pleural effusion, pericardial effusion, acute cholecystitis, distal ileum fistula, blood transfusion, non-invasive ventilation and nephrostomy. She also presented various complications such as intestinal ischemia, surgical wound infection, respiratory insufficiency and hepatic dysfunction.

The patient started with piperacillin, tazobactam and vancomycin, then imipenem with vancomycin. 7 weeks later, the patient was disconnected from the non-invasive ventilation, with no intercurrences and slowly evolving to improve. Two weeks after that she started oral feeding, with associated k108 tube for better caloric income. The patient was treated with antibiotics for 8 days (Colistine), 35 days (Linezolid), 26 days (Ciprofloxacin) and was suspended from the parenteral nutrition since 10 weeks of admission.

In the context of sepsis in abdominal focus with SOFA 2, tomography showed delayed right renal excretion associated with retropielocalicial dilation. Three days later the abdominal fluid was aspirated and passed tests revealing: Creatinine (urine) 37 mg/dL, Urea 819 mg/dL, Sodium 110 mmol/L and Potassium 22 mmol/L. In addition to culture of abdominal fluid showing besides the *Klebsiella pneumoniae*, the presence of *Enterococcus faecium* justifies the exchange of Imipenem to Linezolid[2].

Conclusion

Since the case qualifies as a rare occasion of primary peritonitis with rapid evolution to abdominal sepsis in a healthy individual, it stands to question what were the factors involved. Moreover, it questions what would be the gold-standard treatments to minimize the need for additional procedures, and their complications.

References

1. Fátima Kotleski Thomaz de Lima A., Prevedello Franco R. - Acta Veterinaria Brasilica: SÍNDROME DA RESPOSTA INFLAMATÓRIA SISTÊMICA (SRIS), UM DESAFIO DIAGNÓSTICO, v.3, n.4, p.123-131, 2010

2. Janssens U, et al. Value of SOFA (Sequential Organ Failure Assessment) score and total maximum SOFA score in 812 patients with acute cardiovascular disorders [abstract]. Crit Care 2001;5(Suppl 1):P225.

### P21 Impact of adenosine analog treatment on alveolar macrophage function in mice infected with *Klebsiella pneumoniae* B5055 induced pneumonia

#### Vijay Kumar, Sanjay Chhibber

##### Department of Microbiology, Panjab University, Chandigarh, India

###### **Correspondence:** Vijay Kumar (vij_tox@yahoo.com)

Background

Adenosine is considered as a potent metabokine with potential immunoregulatory function generated during acute inflammatory and infectious conditions. It is produced during conditions causing metabolic stress including hypoxia and inflammation. In the current study, we have investigated the effect of 2-cholroadenosine (2-CADO) on the pulmonary innate immune response including macrophage function in mice infected with *Klebsiella pneumoniae* B5055-induced pneumonia.

Materials and Methods

Acute lung infection/Pneumonia leading to the development of acute lung inflammation or injury (ALI) was induced by intranasal instillation of *K. pneumoniae* B5055 into mice. Subsequently, mice were treated with 2-CADO (10 μg/kg/day/iv) using a treatment schedule. Mice were observed for mortality and other inflammatory damage. Various proinflammatory cytokines (TNF-alpha and IL-1) and anti-inflammatory cytokines including IL-10 were determined by ELISA. Macrophage functions were evaluated in terms of Hydrogen peroxide release, macrophage spreading, and macrophage phagocytic potential. Neutrophil infiltration into the lungs was also evaluated by histopathologic examination of lungs, and myeloperoxidase (MPO) estimation in bronchoalveolar lavage fluid (BALF).

Results

No mortality was observed in both the control and treated groups. 2-CADO treatment modulated the pro-inflammatory function of alveolar macrophages by significantly (p≤0.05) decreasing their phagocytic activity (both phagocytic uptake and intracellular killing of the pathogen), nitric oxide (NO) and hydrogen peroxide (H2O2) release. 2-CADO also significantly (p≤0.05) decreased the neutrophil infiltration into the lungs. Levels of pro-inflammatory cytokines (IL-1α and TNF-α) were decreased significantly (p≤0.05) decreased. However, levels of IL-10 were found to be significantly (p≤0.05) elevated.

Conclusions

Adenosine, a metabokine has promising immunomodulatory action during Gram-negative bacterial pneumonia and associated inflammation.

### P22 Incidence and impact of sepsis and septic shock after subarachnoid hemorrhage

#### Bruno Gonçalves^1^, Pedro Kurtz^1^, Ricardo Turon^1^, Fabio Miranda^1^, Marco Prazeres^1^, Thayana Santos^1^, Fernando Bozza^2^, Cássia Righy^1^

##### ^1^Paulo Niemeyer State Brain Institute, Intensive Care Unit, Rio de Janeiro, Brazil; ^2^Oswaldo Cruz Foundation, National Institute of Infectology, Rio de Janeiro, Brazil

###### **Correspondence:** Bruno Gonçalves (brunogs@gmail.com)

Background

Aneurysmal subarachnoid hemorrhage (SAH) is an acute cerebrovascular disease that can lead to devastating consequences, including high mortality as well as long-term functional impairment among survivors [1]. Approximately 30% of patients hospitalized with SAH will develop some type of nosocomial infection [2]. The diagnosis of sepsis is difficult in patients with SAH due to the high incidence of early systemic inflammatory response syndrome (SIRS), due to the primary bleed, and the lack of reliable biomarkers that can be used to differentiate between SIRS and sepsis [3,4]. Therefore, there is a risk of sepsis misdiagnosis, which can potentially lead to under and overtreatment, in SAH patients. Our objective was to define the incidence of sepsis and septic shock, diagnosed prospectively with new sepsis criteria, and its impact on mortality and functional out-comes of patients with SAH.

Materials and Methods

We prospectively included all adult patients (≥ 18 years) admitted to the Neu-rological Intensive Care Unit (ICU) of the Paulo Niemeyer State Brain Institute (Rio de Janeiro, Brazil) with aneurysmal SAH between April 2016 and May 2018. Daily clinical and laboratory follow-up were analyzed during the first 14 days of hospitalization or up to ICU discharge. Main outcome was the functional outcome, using the Modified Rankin Scale (mRs), prospectively assessed at hospital discharge and after 3, 6 and 12 months.

Results

148 patients were enrolled in the study. 56 (38%) developed 60 infectious events. SIRS was present in 82% and sepsis or septic shock in 28%. In-hospital mortality was 17% Multivariate analysis revealed that death or functional dependence (defined as mRs 4-6) at hospital discharge were independently associated with sepsis/septic shock (OR 3.4, 95% CI 1.16 – 9.96, p = 0.026) (Table 1). SIRS was not independently associated with poor outcome. Mortality, on long-term follow-up, on septic patients was 52.5%, and on non-septic, 16%. Figure 1 shows the Kaplan-Meier survival curve. Figure 2 shows long-term functional outcomes both groups

Conclusions

Sepsis plays a significant role on the outcome of patients with SAH, affecting both mortality and long-term functional outcomes. Combining high-level neurocritical care management of neurological complications and optimal diagnosis and management of sepsis and septic shock may effectively reduce secondary brain injury and improve patients’ outcomes after SAH, especially in low- and middle-income countries.

References

1. Okazaki T, Kuroda Y: Aneurysmal subarachnoid hemorrhage: intensive care for improving neurological outcome. J Intensive Care. 2018;6:28.

2. Festic E, Siegel J, Stritt M, Freeman WD: The utility of serum procalcitonin in distinguishing systemic inflammatory response syndrome from infection after aneurysmal subarachnoid hemorrhage. Neurocrit Care. 2014 Jun;20(3):375–381.

3. Oconnor E, Venkatesh B, Mashongonyika C, Lipman J, Hall J, Thomas P: Serum procalcitonin and C-reactive protein as markers of sepsis and outcome in patients with neurotrauma and subarachnoid haemorrhage. Anaesth Intensive Care. 2004 Aug;32(4):465–470.

4. Connolly ES, Rabinstein AA, Carhuapoma JR, Derdeyn CP, Dion J, Higashida RT, et al: Guidelines for the management of aneurysmal subarachnoid hemorrhage: a guideline for healthcare professionals from the American Heart Association/american Stroke Association. Stroke. 2012 Jun;43(6):1711–1737.

**Table 1 (abstract P22).** Study variables and association with functional outcome (at discharge)

	Unfavorable outcome	Favorable outcome	Univariate analysis	Multivariable analysis	
	(mRankin 4-6) N = 69	(mRankin 0-3) N = 80	P value	OR (95% CI)	P value
Age over 55	37 (53.6%)	30 (37.5%)	0.049	1.04 (1.003 - 1.08)	0.033
Female gender	51 (73.9%)	58 (72.5%)	0.846		
Infection	42 (60.9%)	14 (17.5%)	< 0.0001		
Pneumonia•	23 (33.3%)	4 (5%)	< 0.0001		
SIRS	65 (94.2%)	57 (71.2%)	0.0006		
Sepsis/Septic shock	34 (49.3%)	7 (8.8%)	< 0.0001	3.4 (1.16 - 9.96)	0.026
WFNS 4-5	38 (55.1%)	9 (11.3%)	< 0.0001	4.66 (1.69 - 12.88)	0.003
mFisher 3-4	59 (85.5%)	54 (67.5%)	0.01		
Hydrocephalus	36 (52.2%)	9 (11.3%)	< 0.0001	4.55 (1.61 - 12.85)	0.004
Rebleeding	4 (5.8%)	5 (6.3%)	1		
Vasospasm	34 (49.3%)	21 (26.3%)	0.004		
Post-op deterioration	28 (40.6%)	19 (23.8%)	0.027		
DCI	38 (55.1%)	9 (11.3%)	< 0.0001	3.86 (1.39 - 10.74)	0.01

*mRankin* modified Rankin scale, *SIRS* Systemic inflammatory response syndrome, *WFNS* World Federation of Neurological Surgeons, *mFIsher* modified Fisher scale, *DCI* Delayed cerebral ischemia

*Both ventilator-associated pneumonia and nosocomial pneumonia

**Fig. 1 (abstract P22).**
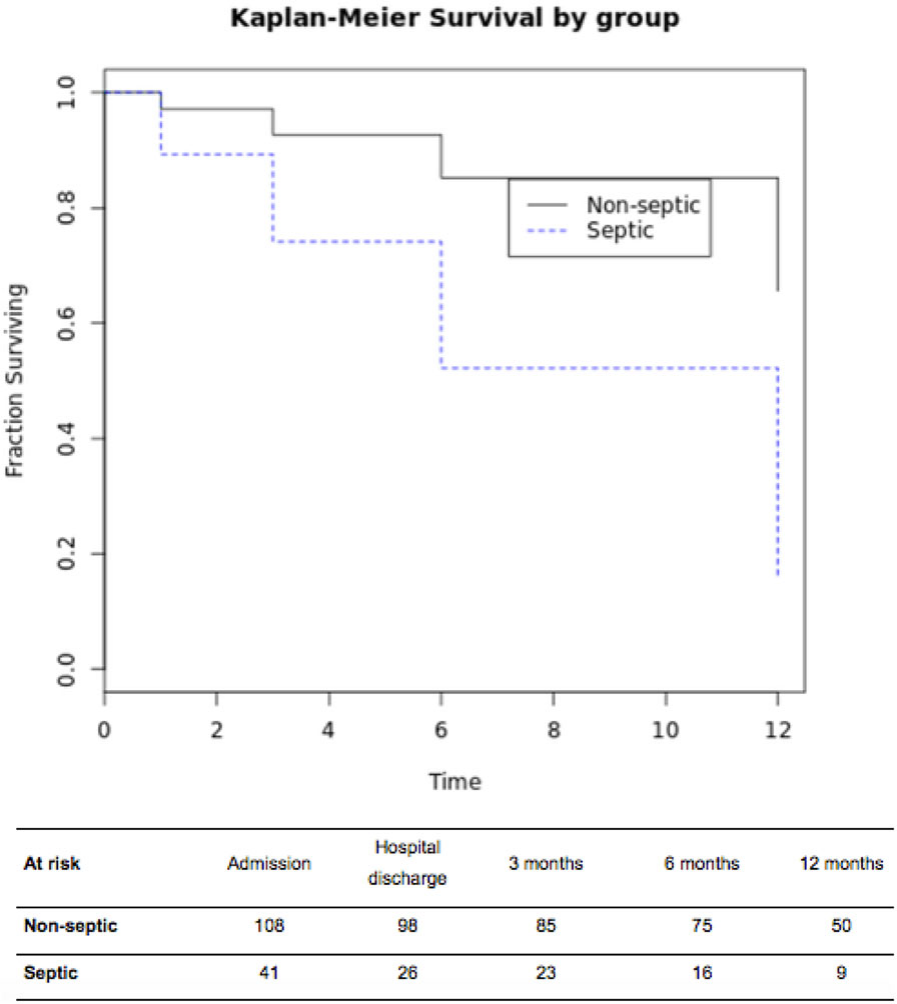
Kaplan-Meier survival curves of septic × non-septic patients with SAH. Log Rank test p<0.0001. Time to death or last follow-up in months

**Fig. 2 (abstract P22).**
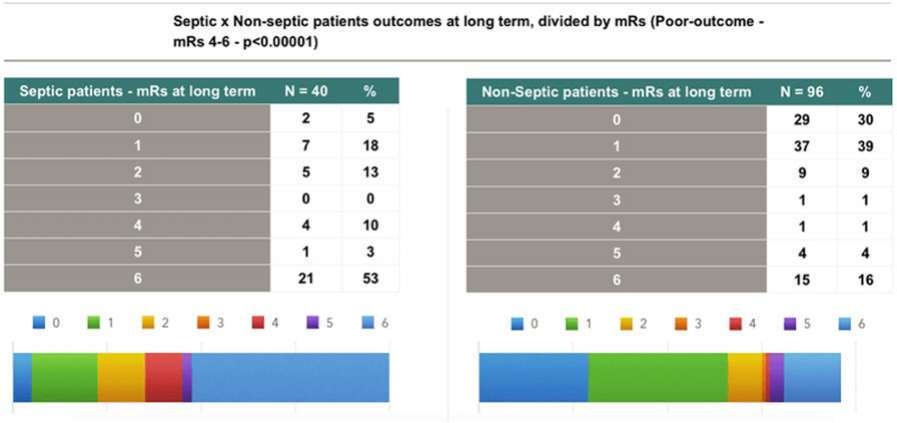
Long-term outcomes on septic × non-septic patients

### P23 Vasoactive drug requirement in sepsis is related to insulin receptor isoform A expression

#### Larissa Aleman^1^, Cecilia Luengo^2^, Marcial Cariqueo^2^, Verónica Rojas^2^, Cristian Espinoza^2^, Julia Guerrero^1,2^

##### ^1^Programa Disciplinario de Fisiología y Biofísica- ICBM- Facultad de Medicina, Universidad de Chile, Santiago, Chile; ^2^Unidad Paciente Critico- Departamento Medicina Interna Hospital Clínico, Universidad de Chile, Santiago, Chile

###### **Correspondence:** Julia Guerrero (jguerrero@med.uchile.cl)

Background

Altered microvascular tone contributes to distributive shock and many evidences supports endothelial damage role on organ dysfunction. Vasopressor drugs requirement is related to vascular reactivity [1]. In other hands, it’s known that altered insulin signaling by differences on its receptor isoforms expression, specifically insulin receptor-A (IR-A) isoform, is related to endothelial dysfunction [2,3]. To our knowledge, there is no previous report of IR-A expression in sepsis.

Materials and Methods

We describe two septic shock (SS) patients, and the association between vasopressor drug requirement and IR-A mRNA expression on mononuclear blood cell at first, second/third and fifth/seventh days after admission. We choose studied IR at monocytes because they are a known model to study IR in humans[4]. IR-A mRNA expression assessed by quantitative polymerase chain reaction and it was as fold of changes by ΔΔCt method analysis.

Results

62 years male (Patient A) and 52 years female (Patient B) with SS criteria of abdominal focus. In both cases, besides antibiotic therapy, crystalloids solutions and vasopressor support to maintain PAM above 65mmHg were necessary. In patient A, the vasopressor requirements were higher at the admission and progressively decreased until its suspension at second day, and the IR-A expression at the beginning was 15,6x and it was progressive decreasing until 1,9x. Patient B required less vasopressor than patient A, but they were necessary for more days. Interestingly, IR-A expression of patient B was lower at the beginning (4,5x) but it remained elevated throughout the observation time (21,5x) as well as vasopressor support.

Conclusions

This is the first report of a plausible association between IR-A expression and vasoactive drugs requirements in SS, probably secondary to endothelial dysfunction. The alternative splicing of exon 11 human IR gene generates two isoforms [5] and, while alterations on IR-B expression are associated to metabolic processes, IR-A upregulated expression has been related to alterations on vascular function [6]. Although IR-A underlying mechanisms are poorly understood, it has been associated to gestational diabetes mellitus endothelial dysfunction [2,3] and cancer angiogenesis [7]. This report shown a higher IR-A expression seems to be associated to higher need for vasopressors. Further research is necessary to establish the IR-A role on vascular reactivity in sepsis.

Acknowledgements

This study was supported by Oficina de Apoyo a la Investigación Clínica (OAIC 929/17) del Hospital Clínico Universidad de Chile. The authors are grateful to Mrs. Rosario Flores and Susana Cañas for its technical assistance. The authors declare no competing interests

References

1. Aird, W. C. The role of the endothelium in severe sepsis and multiple organ dysfunction syndrome. Blood 2003, 101: 3765-3777

2. Westermeier F, Salomon C, González M, et al. Insulin restores gestational diabetes mellitus‐reduced adenosine transport involving differential expression of insulin receptor isoforms in human umbilical vein endothelium. Diabetes 2011, 60: 1677–1687

3. Guzmán‐Gutiérrez E, Westermeier F, Salomón C, et al. Insulin‐increased L‐arginine transport requires A2A adenosine receptors activation in human umbilical vein endothelium. PLoS One 2012, 7: e41705

4. Beck-Nielsen H, Pedersen O, Kragballe K, Sorensen NS. The monocyte as a model for the study of insulin receptors in man. Diabetologia 1077,13: 563-569

5. Belfiore A, Malaguarnera R, Vella V, et al. Insulin Receptor Isoforms in Physiology and Disease: An Updated View. Endocr Rev. 2017, 38:379-431

6. Westermeier F, Sáez T, Arroyo P et al. Insulin receptor isoforms: an integrated view focused on gestational diabetes mellitus. Diabetes Metab Res Rev 2016, 32: 350–365

7. Nowak-Sliwinka P, van Beijnum JR, Huijbers EJM et al. Oncofetal insulin receptor isoform A marks the tumour endothelium: an underestimated pathway during tumour angiogenesis and angiostatic treatment. Br J Cancer 2019, 120: 218-228

### P24 Fulminant septic shock associated with *Herpes Simplex Virus* encephalitis

#### Sandra Fernandez-Caballero, Maria Perez-Herrero

##### Department of Anesthesiology. Hospital Clínico Universitario de Valladolid. Valladolid, Spain

###### **Correspondence:** Sandra Fernandez-Caballero (sandrafercab@hotmail.com)

Background

*Herpes simplex virus-1* (HSV-1) is the most common infectious cause of sporadic encephalitis in older adults and young children. There are fatal prognostic factors like lower level of consciousness at presentation, older age, severe comorbid disease, immunodepression and delay in start of treatment. This is a potentially lethal illness with a high risk of morbidity and death and it is necessary to do an early diagnosis with a good clinical history, examination, Computed tomography (CT) scan and/or Magnetic Resonance Imaging (MRI), and lumbar puncture. Prompt treatment using acyclovir therapy can give a better prognosis, however, sometimes the disease tends to progress rapidly causing septic shock and multiorgan failure.

Materials and Methods

A 71-year-old woman was admitted to the Emergency department presenting with a two-day history of fever and speech impairment. She was previously healthy with no past medical history. On initial examination she was disorientated in time, space and person and her speech was unintelligible. Computed tomography CT) scan showed left temporal lobe hypodense lesion (Fig.1) indicating viral encephalitis. After few hours, she started to be drowsy with low Glasgow Coma Scale (GCS) requiring intubation and was transferred to the High Dependency Unit (HDU). Her chest X-ray was suggestive of aspiration pneumonia (Fig.2). Due the possibility of viral encephalitis and pneumonia, early empiric antibiotics and acyclovir, were started. Lumbar puncture was performed and the cerebrospinal fluid (CFS) analysis revealed lymphocytic pleocitosis, elevated protein, normal glucose and positive polymerase chain reaction (PCR) for *herpes simplex virus* (HSV)-1.

Results

The patient continued to deteriorate and septic shock was suspected. She was tachycardic (heart rate 140/min) and had severe arterial hypotension (blood pressure 60/30) despite aggressive intravenous fluid resuscitation and norepinephrine. Arterial blood gas revealed metabolic acidosis with hyperlactatemia. She had an ongoing progressive organ dysfunction with coagulopathy, acute renal failure and severe hypoxia, and was unresponsive to resuscitation measures and high doses of amines, finishing with completely irreversible organ failure.

Conclusions

*Herpes simplex virus* is one of the most common causes of encephalitis. It is important to suspect, diagnose and treat as soon as possible in order to reduce the mortality and morbidity rates. The use of dexamethasone is unclear but can be considered when there is a progression even when acyclovir has been started. Despite the early treatment, this severe infection has the potential to cause severe neuropsychiatric deficits, or septic shock and multiorgan failure with death, like in our case report.

Consent

Written, informed consent for publication was obtained from the patient for publication of this case report.

**Fig. 1 (abstract P24).**
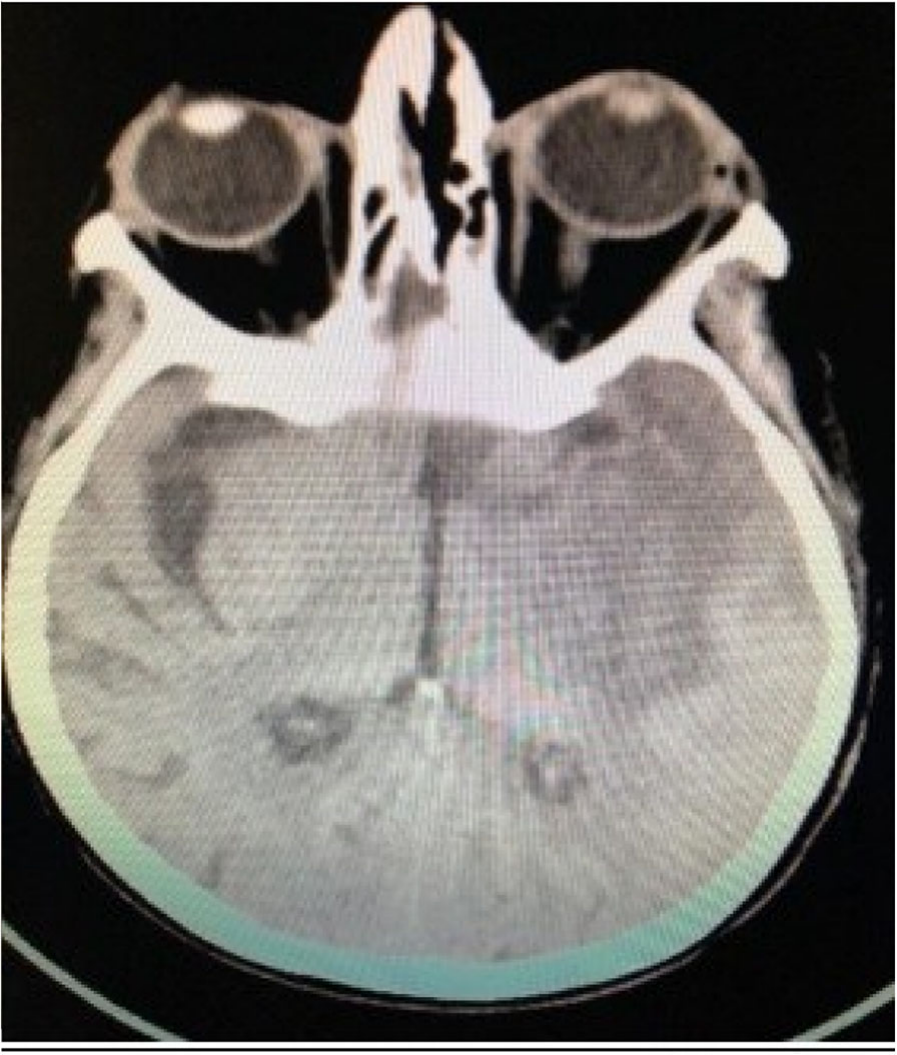
See text for description

**Fig. 2 (abstract P24).**
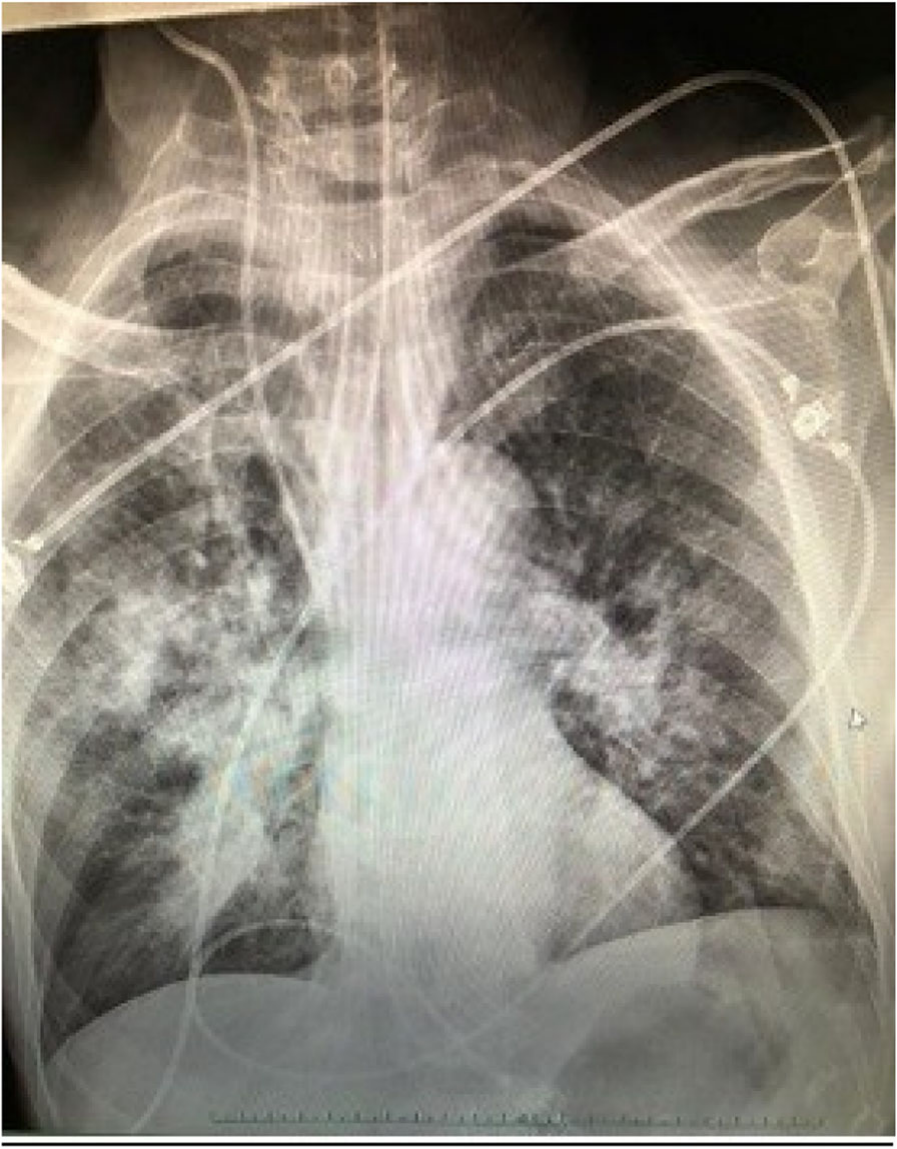
See text for description

### P25 *Klebsiella pneumoniae* pneumonia complicated with an abscess and empyema in an alcoholic

#### Sandra Fernandez-Caballero, Maria Perez-Herrero

##### Department of Anesthesiology. Hospital Clínico Universitario de Valladolid. Valladolid, Spain

###### **Correspondence:** Sandra Fernandez-Caballero (sandrafercab@hotmail.com)

Background

*Klebsiella pneumoniae* is a common bacterial pathogen that can cause different diseases like pneumonia, bloodstream and urinary tract infections, liver abscess, necrotizing fasciitis, meningitis, sepsis. It is an unusual cause of community acquired pneumonia except in alcoholics. An important problem is the increasing number of strains resistant to antibiotics.

Materials and Methods

A 63-year-old male, with a history of alcohol abuse, presented to the Emergency Department with fever, dyspnea and productive cough with greenish sputum. He complained of nausea and vomiting for the last four days. Clinical examination revealed a conscious, oriented but distressed patient. He was clammy, sweaty, tachycardic (128 beats/min), hypotensive (85/50 mmHg), tachypneic (30 breaths/min), and his oxygen saturation was 88%. Lung auscultation showed crackles and his chest X-ray an extensive right upper lobe consolidation (Fig.1). Arterial blood gas displayed a mixed acidosis with pO2 56mmHg. Blood, urine, and sputum cultures were obtained and empiric antibiotics were initiated (piperacillin-tazobactam and azithromycin). He was immediately admitted to the High Dependency Unit (HDU) with the diagnosis of pneumonia and sepsis. High flow nasal cannula was started but the patient was using accessory muscles and showed a gradually worsening shortness of breath, requiring intubation and connection to mechanical ventilation. Blood test results revealed leucopenia, lactic acidosis, acute renal failure and high C-Reactive Protein (CRP).

Results

During his admission, the patient was hypoxic and in septic shock, requiring high FiO2 and fluid resuscitation plus vaso-active amine infusion, trying to achieve hemodynamic stabilization. Thoracic computed tomography (CT) scan exhibited the presence of extensive alveolar consolidation in right upper, middle and lower lobe. A *Klebsiella pneumoniae* was detected in sputum and blood cultures, and ceftazidime-avibactam was started. Even though the correct treatment was used, the patient was not improving. After 10 days, a second CT demonstrated worsening consolidation, abscess in right upper lobe and empyema (Fig.2). Progressive septic shock led to multiple organ failure not responding to the intensive management.

Consent

Written, informed consent for publication was obtained from the patient for publication of this case report.

Conclusions

*Klebsiella pneumoniae* is an opportunistic pathogen that can cause different nosocomial infections and must be considered in alcoholic patients with severe pneumonia. This pathogen always requires prior treatment due his virulence and multiple antimicrobial resistance. We have to be very cautious with invasive infections produced by this microorganism that are strongly associated with immunocompromised populations.

**Fig. 1 (abstract P25).**
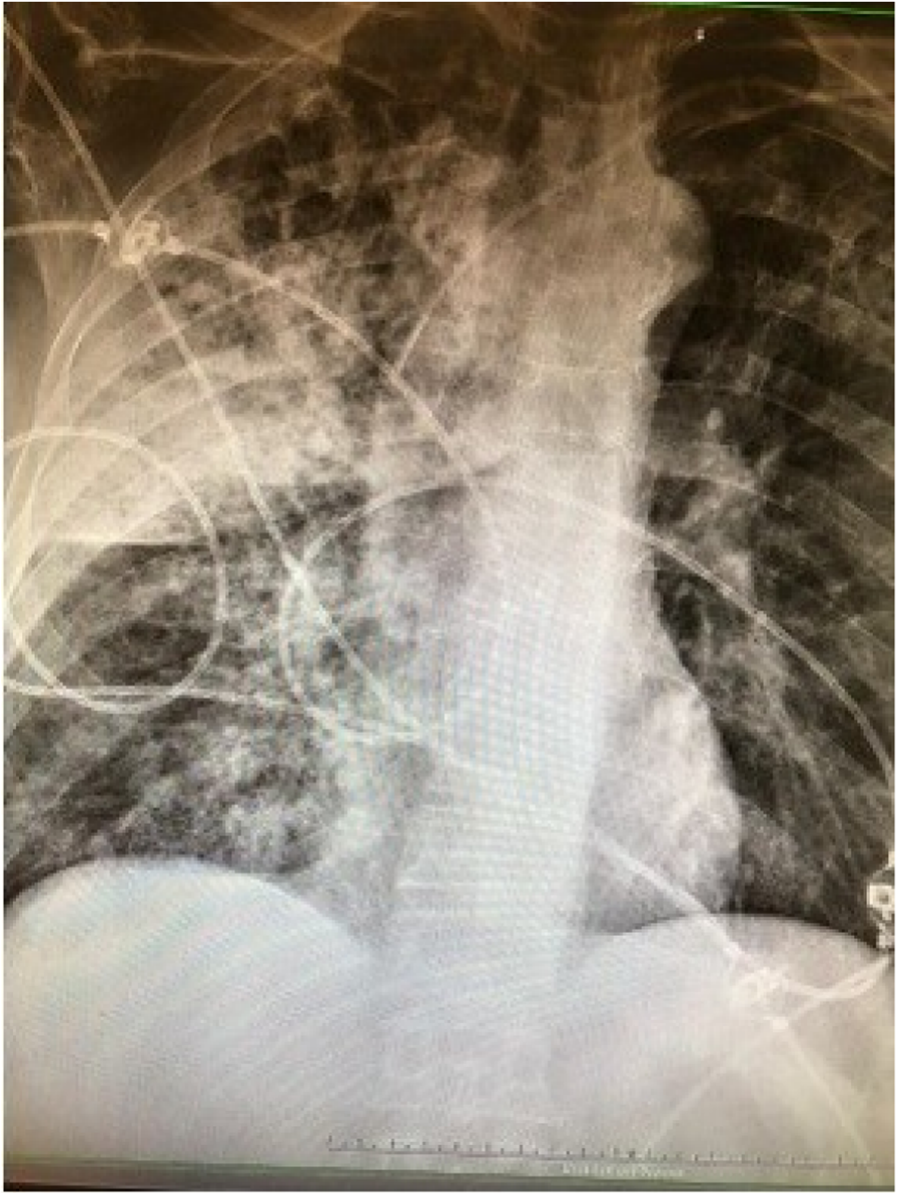
See text for description

**Fig. 2 (abstract P25).**
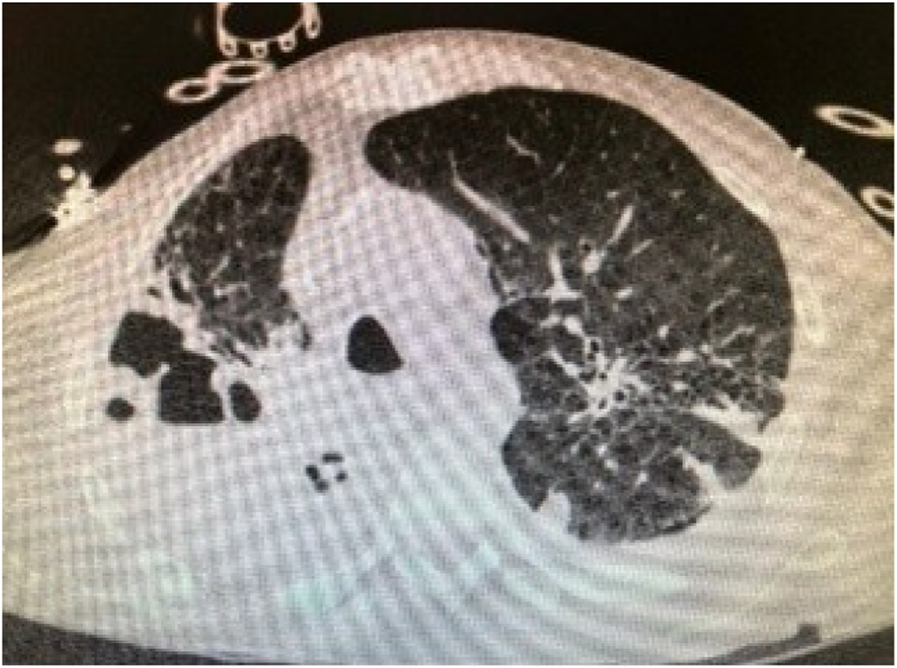
See text for description

### P26 Changes in central venous pressure during fluid challenge have limited valued to guide fluid therapy

#### Priscilla S Oliveira, Antônio T Bafi, Flávia R Machado, Flávio GR Freitas

##### Anesthesiology, Pain and Intensive Care Department, Federal University of São Paulo, São Paulo, Brazil

###### **Correspondence:** Flávia R Machado (frmachado@unifesp.br)

Background

Static values of central venous pressure (CVP) have limitations to guide fluid management although dynamic changes are considered useful [1-3]. We evaluated if CVP changes after a fluid challenge and the baseline cyclic respiratory variation in CPV amplitude curve (ΔCVP) could discriminate responders from non-responders.

Materials and Methods

This is a prospective, observational study conducted in two mixed intensive care. We included adult patients under mechanical ventilation, adequately sedated and with acute circulatory failure. They received a fluid challenge with crystalloids (Ringer's lactate or sodium chloride 0.9% solution, 500 mL) infused over 15 minutes. We determined the CVP at baseline (CVPT0) and the changes at 5 (∆CVPT5), 10 (∆CVPT10) and 15 (∆CVPT15) minutes during fluid infusion. We also measured ΔCVP at baseline. Fluid responsiveness was defined by a cardiac index increased ≥ 15%.

Results

We included 30 patients (11 responders, 19 non-responders). There was a significant increase in mean CVP over time in both responders and non-responders (0.12 mmHg; standard error: 0.02; P<0.001), although there were no statistically significant differences between the groups in the mean CVP (-2.7 mmHg; standard error: 1.37; p=0.06) and in the mean CVP changes up to 15 minutes (-0.0 mmHg; standard error: 0.03; p=0.25). The cardiac index did not correlate with the changes in CVP after the fluid challenge (R=-0.25, p value = 0.18). The CVPT0 and the changes after fluid challenge in all three timepoints did not adequately predict fluid responsiveness (CVPT0 - AUC: 0.70 (95%CI: 0.49 - 0.90), ∆CVPT5 - AUC: 0.78 (95%CI:0.57 - 0.99), ∆CVPT10 - AUC: 0.63 (95%CI:0.39 - 0.88), ∆CVPT15 - AUC: 0.68 ((95%CI: 0.45 - 0.92)). The ΔCVP at baseline also had poor performance (AUC: 0.70 (95%CI: 0.50 - 0.91).

Conclusions

Dynamic changes in CVP have limited value to guide fluid management. Changes in CVP up to 15 min after fluid infusion and ΔCVP at baseline should not be used as a marker of fluid responsiveness in adult patients under mechanical ventilation.

Conflicts of interest

The authors have no conflict of interest

Funding

This study had no specific support

References

1. Marik P, Bellomo R. A rational approach to fluid therapy in sepsis. British Journal of Anaesthesia 2016;116(3):339-349.

2. Eskesen TG, Wetterslev M, Perner A. Systematic review including re-analyses of 1148 individual data sets of central venous pressure as a predictor of fluid responsiveness. Intensive Care Medicine 2016;42(3):324-332.

3. Pinsky MR, Kellum JA, Bellomo R. Central venous pressure is a stopping rule, not a target of fluid resuscitation. Critical Care and Resuscitation 2014;16(4):245-246.

### P27 Components of fluid balance in patients with septic shock: what differentiates survivors from non-survivors?

#### Maria AS Silva, Andréia L Silva, Antônio T Bafi, Eduardo S Pacheco, Nelma LM Cruz, Bianca S Svicero, Flávia R Machado, Flávio G R Freitas

##### Anesthesiology, Pain and Intensive Care Department, Universidade Federal de São Paulo, São Paulo, Brazil Intensive Care Unit, Hospital Sepaco, São Paulo, Brazil

###### **Correspondence:** Flávio G R Freitas (flaviogrf@yahoo.com.br)

Background

A positive fluid balance is associated with worst outcomes in critically ill patients. Our objective was to identify the main components of fluid balance (FB) in patient with septic shock and to compare the proportion of these components between survivors and non-survivors.

Materials and Methods

A prospective observational study including adult patients with septic shock (Sepsis 2.0 criteria) admitted to an adult intensive care units (ICU) in two Brazilian hospitals. Fluid intake was calculated as the sum of intravenous, oral and enteral fluids and fluid loss as the sum of diuresis, ultrafiltration and drains losses. Fluid balance (FB) was determined as the difference between fluid intake and fluid loss. Based on patients’ charts, we obtained data from 6 h before vasopressor drugs start, as well as on each 24 hours after, up to 72 hours.

Results

We included 139 patients between May 2016 and January 2017. Hospital mortality was 53%. In the whole study period, FB was higher in non-survivors (5856 (3560-9245)ml) than in survivors (3723 (2347-5780) ml), p <0.01, mainly due to a lower fluid loss (non-survivors: 4105 (2740-6121) ml; survivors: 5505 (4045-7050) ml, p <0.01). Fluid intake were slightly higher in non-survivors (10391 (8547-12969)ml) than in survivors (9651 (7979-11007) ml), p = 0.046. In the whole period, non-survivors received more vasoactive drugs (905 (357-1957)ml) than survivors (302 (133-737) ml), p <0.01, but received less enteral intake (non-survivors: 1296 (167-3030); survivors:2725 (1610-4160) ml, p = 0.01). The difference in vasopressor and nutrition were also significant in the first 24 hours. Fluid intake for maintenance fluid therapy, fluid challenges, dilution of antibiotics, blood components and dilution of other medications were similar between the two groups in the whole period and in the first 24 hours after starting vasopressor.

Conclusions

FB was higher in non-survivors, mainly due to lower fluids output. They received similar fluids as fluid challenge and maintenance therapy. However, gained more vasoactive drugs and less enteral intake.

